# Effects of miR-101-3p on goat granulosa cells *in vitro* and ovarian development *in vivo* via *STC1*

**DOI:** 10.1186/s40104-020-00506-6

**Published:** 2020-10-14

**Authors:** Xiaopeng An, Haidong Ma, Yuhan Liu, Fu Li, Yuxuan Song, Guang Li, Yueyu Bai, Binyun Cao

**Affiliations:** 1 College of Animal Science and Technology, Northwest A&F University, No. 22 Xinong Road, Yangling, Shaanxi 712100 P.R. China; 2 College of Biological Science and Engineering, Shaanxi University and Technology, Hanzhong, Shaanxi, 723001 P.R. China; 3 Henan Animal Health Supervision Institution, No. 91 Jingsan Road, Zhengzhou, Henan 450008 P.R. China

**Keywords:** Granulosa cells, MiR-101-3p, Ovary, STC1, Transcriptome

## Abstract

**Background:**

MiRNAs act as pivotal post-transcriptional gene mediators in the regulation of diverse biological processes, including proliferation, development and apoptosis. Our previous study has showed that miR-101-3p is differentially expressed in dairy goat ovaries compared single with multiple litters. The objective of this research was to explore the potential function and molecular mechanism of miR-101-3p via its target *STC1* in goat ovarian growth and development.

**Results:**

cDNA libraries were constructed using goat granulosa cells transfected with miR-101-3p mimics and negative control by RNA-sequencing. In total, 142 differentially expressed unigenes (DEGs) were detected between two libraries, including 78 down-regulated and 64 up-regulated genes. GO and KEGG enrichment analysis showed the potential impacts of DEGs on ovarian development. *STC1* was singled out from DEGs for further research owing to it regulates reproductive-related processes.* In vitro*, bioinformatics analysis and 3′-UTR assays confirmed that *STC1* was a target of miR-101-3p. ELISA was performed to detect the estrogen (E2) and progesterone (P4) levels. CCK8, EdU and flow cytometry assays were performed to detect the proliferation and apoptosis of granulosa cells. Results showed that miR-101-3p regulated *STAR, CYP19A1, CYP11A1* and *3β-HSD* steroid hormone synthesis-associated genes by *STC1* depletion, thus promoted E2 and P4 secretions. MiR-101-3p also affected the key protein PI3K, PTEN, AKT and mTOR in PI3K-AKT pathway by *STC1*, thereby suppressing proliferation and promoting apoptosis of granulosa cells. *In vivo*, the distribution and expression levels of miR-101-3p in mouse ovaries were determined through fluorescence *in situ* hybridisation (FISH). Immunohistochemistry results showed that *STC1* expression was suppressed in mouse ovaries in miR-101-3p-agonist and siRNA-STC1 groups. Small and stunted ovarian fragments, decreased numbers of follicles at diverse stages were observed using Hematoxylin-eosin (HE) staining, thereby showing unusual ovarian development after miR-101-3p overexpression or *STC1* depletion. Inhibition of miR-101-3p manifested opposite results.

**Conclusions:**

Taken together, our results demonstrated a regulatory mechanism of miR-101-3p via *STC1* in goat granulosa cells, and offered the first *in vivo *example of miR-101-3p and *STC1* functions required for ovarian development.

## Introduction

In mammals, folliculogenesis and oogenesis proceed in parallel within the ovary [1]. Ovarian follicle consists of an oocyte which surrounded by theca and granulosa cells, and is closely related to ovulation, fertilization, implantation and embryo growth [2]. Granulosa cells preserve and breed oocytes as well as secrete steroid hormones such as estrogen and progesterone, furnishing a crucial microenvironment for follicular growth [3]. Proliferation and differentiation of granulosa cells is elementary for development of follicle and oocyte, ovulation and luteinization, while apoptotic cell death performs the mechanism of ovarian follicle atresia. Thus granulosa cells are supposed to maintain ovarian function [2, 3].

MiRNAs are a series of conserved noncoding RNA molecules with 18–25 nucleotides (nts) in length [4]. They can elicit mRNA degradation or translational suppression by pairing with the 3′-untranslated region (UTR) of their target genes. Subsequently miRNAs reduce gene expression at the post-transcriptional level and undergo a series of physiological events including development, growth, proliferation and apoptosis [5, 6]. MiRNAs also have vital functions on regulating ovarian growth and development. For example, miR-224 [7], miR-383 [8] and miR-378 [9] regulate steroid hormones secretions in granulosa cells, while miR-145 [10], miR-23a [11] and miR-26b [12] take effects on proliferation and apoptosis of granulosa cells by binding to target genes. MiR-21 [13], miR-503 [14], miR-125b and miR-145 [15] participate in ovulation, follicular-luteal transformation and luteal process. Based on our prevenient work, we selected miR-101-3p for further research owing to its differentially expression in dairy goat ovaries between single and multiple litter sizes (not published). It’s reported that miR-101-3p is a tumour eliminator and implicates a range of tumour-related biological processes. For example, miR-101 suppresses cell proliferation and facilitates cell apoptosis by inhibiting *mTOR* in Saos-2 cells [16], promotes Bcl2-regulated apoptosis by *RLIP76* in prostate cancer cells [17], represses tumour growth and migration by *ROCK1* in osteosarcoma cells [18]. However, the functions of miR-101-3p on goat ovaries remain comparatively uncharacterised.

Bioinformatics analysis finds that *STC1* is a potential target of miR-101-3p. *STC1* is a member of stanniocalcin (STC) family, and the other closely pertinent orthologue is *STC2* [19]. STC, a glycoprotein hormone identified in bony fish, can modulate calcium and phosphate levels which produced in the corpuscles of Stannius [20]. In several mammalian tissues, *STC1* and *STC2* emerge as paracrine/autocrine rather than endocrine compared with their topical glandular expression in fish, thus modulating mineral metabolism [21]. *STC1* regulates abundant vital biological processes such as cellular activities, lactation, pregnancy and organogenesis. For example, the elevated expression of *STC1* is discovered in breast carcinomas and ovarian cancer, which means *STC1* may act as a carcinogenesis factor [22]. The activation of *STC1* is observed during gestation and lactation in mouse ovaries, suggesting a gestational and nursing state function [23]. The role of *STC1* in ovaries is also enhanced by identifying the subcellular luteal cell targets, cholesterol or lipid storage droplets (steroidogenic active regions) [24]. *STC1* shows inhibitory effects on FSH-, LH- and hCG-stimulated progesterone synthesis in rat granulosa cells and bovine luteal cells [25, 26]. Whereas, it remains indistinct whether *STC1* is capable of taking effects on goat ovaries.

In present study, we have accomplished the global transcriptional analysis of miR-101-3p overexpressed goat granulosa cells and identified the DEGs by RNA-seqencing (RNA-Seq) method. From the down-regulated DEGs we selected *STC1*, also a potential target of miR-101-3p for further studies on account of its involvement in ovarian function. Then we evaluated the regulatory roles and molecular mechanism of miR-101-3p on steroid hormone synthesis, cell proliferation and apoptosis via targeting *STC1* in goat granulosa cells *in vitro*. Finally we detected the effects of miR-101-3p and *STC1* on mouse ovaries *in vivo*. The current study used *in vitro* and *in vivo* models to find out how miR-101-3p and *STC1* function on ovarian development.

## Materials and methods

### Cell culture

The Xinong Saanen dairy goats (1–3 years old, not estrus) in the experimental farm of Northwest A&F University of China were used. The collected ovaries were washed and maintained in PBS with penicillin (100 μg/mL) and streptomycin (100 μg/mL) and then transferred to culture dishes. Goat granulosa cells were released into the medium when the large antral follicles were punctured by hypodermic needles. HEK293T cells were bought from Shanghai Tongwei Company and thawed from liquid nitrogen directly in 37 °C sterile water. Granulosa cells or HEK293T cells were cultivated in DMEM/F12 medium (Gibco, Grand Island, USA) or DMEM (high glucose) medium (Gibco, Grand Island, USA) both supplemented with 10% foetal bovine serum (FBS), penicillin (100 μg/mL) and streptomycin (100 μg/mL) in a humidified atmosphere with 5% CO_2_ at 37 °C.

### PcDNA3.1-STC1 plasmid construction

The CDS regions of STC1 (XM_005684015) were extended using PCR derived from the extracted cDNA of goat granulosa cells. The PCR products were digested and cloned into pMD™19-T vector (TakaRa, Ostu, Japan). Afterwards, *STC1* overexpression plasmids were constructed using the eukaryotic expression pcDNA3.1(+) vector (Thermo Fisher, Shanghai, China) between Hind III and Xho I sites. The entire *STC1* CDS sequences were introduced into the numerous cloning spots of the pcDNA3.1 vector, and the constructs were confirmed through DNA sequencing. The forward and reverse primers of *STC1* were Hind III 5′-CCCAAGCTTAGCAACTTAGCGGAAACT-3′ and Xho I 5′-CCGCTCGAGGCGTAAACACCCTTAAAAC-3′, respectively.

### Transfection and RNA extraction

Granulosa cells (5 × 10^6^ cells/well) were precultured in 6-well plates. The miR-101-3p mimics (miR-101-3p-mi), mimics negative control (NC-mi), miR-101-3p inhibitors (miR-101-3p-in), inhibitors NC (NC-in), siRNA-STC1 (si-STC1), siRNA negative control (NC) (GenePharma, Shanghai, China), pcDNA3.1 and pcDNA3.1-STC1 vectors were transfected into granulosa cells by Lipofectamine 2000 (Invitrogen, Carlsbad, USA). Table S1 shows the sequences. Optimal medium concentration of mimics or inhibitors were used in this study based on the manufacturer’s standards. Briefly, 5 μL miR-101-3p mimics/NC-mi (50 nmol/L) or inhibitors/NC-in (100 nmol/L) or si-STC1/NC (50 nmol/L) with 5 μL Lipofectamine 2000, 5 μg pcDNA3.1-STC1/pcDNA3.1 vectors with 6 μL Lipofectamine 2000, 2.5 μL miR-101-3p-mi and 2.5 μg pcDNA3.1-STC1 vectors with 6 μL Lipofectamine 2000 were diluted in 200 μL Opti-MEM I medium (Gibco, CA, USA) and cultivated for 20 min. Then the medium was replaced within 4–6 h. After 24 or 48 h transfection, the cells were harvested for further detection. RNA was extracted and purified by TRIzol reagent (Invitrogen, CA, USA). Agilent 2100 Bioanalyzer (Agilent Technologies, CA, USA) was used to evaluate RNA concentration and purity. The ratio of the optical densities estimated at 260 and 280 nm was > 1.8 and < 2.0 for all RNA samples.

### Library preparation for sequencing

After goat granulosa cells transfected with miR-101-3p mimics and negative control (NC) for 24 h (each treatment had three repeats), the extracted RNA were used to construct RNA libraries. The optical densities of 260/280 nm in miR-101-3p mimics group were 1.91, 1.95 and 1.90, those of NC group were 1.88, 1.94 and 1.80. Library preparation for sequencing of each experimental sample was instituted based on the manufacturer’s standards. In brief, mRNA was enriched using oligo magnetic beads after total RNA extraction. The purified mRNA was first divided to 200–300 bp fragments with an RNA fragmentation kit. Utilising random hexamer primers and reverse transcriptase, the first-strand cDNA synthesis was accomplished. Afterwards, a tailored second-strand primer and strand synthesis enzyme mix were added, followed by incubation with dNTPs, DNA polymerase I and RNase H to synthesise the second strand. The mixes were subjected to end repair with an End Prep enzyme mix after purification using an Agilent 2100 bioanalyzer, supplemented with adaptor ligation, single A base and agarose gel insulation of 300–400 bp cDNA. The library outcomes were used for sequencing reaction in an Illumina HiSeq™ 2000 platform.

### Read mapping on the goat reference genome and data analysis

The information was converted into sequence data, and the premier image was selected. After the 3′adaptor sequences were filtered, low-quality reads (the percentage of low quality measured with *Q* value of ≤5 was more than 50% in each read) with more than five Ns per 100 bp and superfluous reads were separated. The clean reads were arranged according to the goat reference genome (GCF_001704415.1_ ARS2 _genomic.fa) for convergence by Top Hat v2.0.12.

The correlation among the repeated biological samples was checked to ensure that the selection of samples was reasonable. The square (R^2^) of Pearson correlation coefficient was used to represent the similarity of expression patterns between samples. R^2^ > 0.92 was considered as the ideal sampling and experimental condition. All the spliced forms of the transcript were determined based on Stringtie, and new transcript regions were found using gffcompare compared with the reference genome. According to the genetic model predicted by Stringtie for each sample, ASprofile software (http://ccb.jhu.edu/software/ASprofile/) was used to classify and count the variable splicing events. InDel sites were obtained by Varscan (version 2.3.7).

### Standardised expression levels of genes and screening of DEGs

Reads per kilobase transcriptome per million mapped reads (RPKM) (RPKM = entire exon reads/mapped reads in millions × exon length in kb) method was used to normalise the gene expression levels. RPKM > 1 was used as the threshold to judge gene expression. Differentially expressed unigenes (DEGs) and their comparative *P*-values were calculated according to previous study [27]. The significance limitation of the *P*-value in numerous tests was fixed on the basis of FDR. Standardised gene expression levels of groups were measured using the fold changes (log_2_ |Ratio|) by DESeq (version 1.18.0). Finally, the standards of (i) *P*-value < 0.05 and (ii) log_2_ |Ratio| > 1 were utilised to determine the significance of gene expression differences. Volcano and MA plot maps of DEGs were created using the R language ggplots2 software package.

### Gene Ontology and KEGG pathway analysis of DEGs

The DEGs were categorised into molecular function, cellular component and biological process using Gene Ontology (GO) annotation [28]. Hypergeometric detection was implemented to match all DEGs to terms in the database (http://www.geneontology.org/) and to examine the apparently enriched GO terms of DEGs. Next, we used KEGG (http://www.genome.jp/kegg/), an elementary public pathway-correlative database to appraise remarkably enriched signal transduction pathways or metabolic pathways of DEGs [29]. The valuation formula was the same as the value of GO annotation.

### Quantitative real-time PCR (RT-qPCR)

For the mRNA expression level, 0.3 mg of total RNA was synthesized into cDNA using PrimeScripts RT Reagent Kit (TakaRa, Ostu, Japan) according to the manufacturer’s specifications. After reverse transcription, TB Green™ Premix Ex Taq™ II (TakaRa, Ostu, Japan) was used to quantify the relative amount of miR-101-3p and disparate genes by the CFX Connect Real-Time PCR Detection System (Bio-Rad, Hercules, USA). Table S2 shows the validated primers used for RT-qPCR. In brief, the amplification reaction mixtures of 25 μL contained 2 μL cDNA, 12.5 μL SYBR Premix Ex Taq II, 1 μL PCR Forward and Reverse Primers and 2.5 μL ddH_2_O. The reaction conditions were: initial denaturation for 30 s at 95 °C, denaturation for 5 s at 95 °C, followed by 40 cycles of annealing for 30 s at 60 °C and extending for 50 s at 72 °C. At the end of the total runs, a melting-curve analysis (95 °C for 15 s and 60 °C for 1 min at 0.5 °C/5 s until 95 °C) was performed to ensure the specificity of amplification. U6 or *β*-actin was detected as a loading control for miRNA or genes mRNA levels. Each condition was repeated in three wells, all experiments were performed in triplicate. Relative expression was calculated through the 2^−ΔΔCt^ approach.

### Western blot

After each transfection for 48 h, granulosa cells were collected and lysed with the ice-cold RIPA lysis buffer (Bioteke, Beijing, China) supplemented with 0.1 mg/mL PMSF (Solarbio, Beijing, China). The mixtures were centrifuged at 4 °C and proteins were then extracted from the cells. BCA Protein Assay kit (Vazyme Biotech, Nanjing, China) determined the protein concentration. SDS-PAGE (12%) was performed with 40 μg total protein approximately via electrophoresis, after which the proteins were diverted into polyvinylidene difluoride membranes (Merck Millipore, Darmstadt, Germany). After shaking in 5% non-fat dry milk at room temperature for 2 h, the membranes were incubated with primary antibodies at 4 °C overnight and species-specific secondary antibodies for 2 h, which were eventually colour-analysed with a Beyo ECL Star kit (Beyotime, Shanghai, China). The primary antibodies were as follows: HSD3B1 (1:1000, Abcam, London, UK), STAR (1:1000, Abcam, London, UK), CYP11A1 (1:600, Bioss, Beijing, China), TP53 (1:500, BBI, Shanghai, China), CASP3 (1:700, BBI, Shanghai, China), ACTB (1:1000, BBI, Shanghai, China), STC1 (1:700, ABclonal, Wuhan, China), PI3K (1:400, ABclonal, Wuhan, China), mTOR (1:600, ABclonal, Wuhan, China), p-mTOR (1:600, ABclonal, Wuhan, China), AKT (1:600, ABclonal, Wuhan, China), p-AKT (1:400, ABclonal, Wuhan, China), PTEN (1:900, ABclonal, Wuhan, China), Bcl-2 (1:500, ABclonal, Wuhan, China), BAX (1:500, ABclonal, Wuhan, China), CCND1 (1:800, ABclonal, Wuhan, China), CDK4 (1:600, ABclonal, Wuhan, China), CCNE1 (1:600, ABclonal, Wuhan, China) and PCNA (1:500, ABclonal, Wuhan, China). The transfection efficiency of miR-101-3p and *STC1* is showed in Fig. S1.

### 3′-UTR luciferase reporter assay

The sequences of *STC1* 3′-UTR containing the predicted target sites of miR-101-3p were amplified from goat genomic DNA through PCR. Afterwards, the PCR fragments were inlet into psiCHECK-2 vectors with particular Xhol I and Not I restriction enzymes for dual-luciferase assay. Forward primer of wild-type (wt) STC1: Xho I 5′-CCGCTCGAGTAAGGTCTAACTGGAATA-3′, reverse primer: Not I 5′-AAATATGCGGCCGCATCTCATACAGGCCCAT-3′. To institute mutated 3′-UTR reporter vectors, the calculated binding sites were mutated by site-directed mutagenesis with specific primers. Forward primer of mutant (mu)-type STC1: Xho I 5′-GGCTCGAGTCCTCAGTGTCTAATTTCCT-3′, reverse primer: Not I 5′-TAGCGGCCGCCTTTGTTTAAGCAGAGTCCT-3′. HEK293T cells were seeded in a 48-well plate and then co-transfected 10 pmol miR-101-3p-mi/NC-mi or miR-101-3p-in/NC-in with 0.33 mg wt-/mu- luciferase reporter gene plasmids using Lipofectamine 2000 for 48 h (*n* = 3). The renilla and firefly luciferase activities were evaluated using a Dual-Glo luciferase assay system (Promega, Madison, USA) with Thermo Scientific Varioskan Flash (Thermo Scientific, Shanghai, China). Firefly luciferase activity was normalized to renilla luciferase activity.

### ELISA

Granulosa cells were seeded and harvested in 6-well plates when post-transfected in 24 h. Cell-free supernatants were assembled and used in the evaluation of oestrogen (E2) and progesterone (P4) production with an enzyme-linked immunosorbent assay (ELISA) kit (Zhenke, Shanghai, China). Based on the kit specifications, the absorbance at 450 nm with 50 μL supernatants was set using an Epoch microplate reader (Biotek, Winooski, USA). The corresponding concentrations of the samples were calculated using the Equation from the linear regression of the standard curve was obtained and then calculated corresponding concentrations of the samples. The mean intra- and inter-assay variable coefficient values were less than 15%, and the sensitivity of kits was 1 pmol/mL.

### CCK8 assay

The viability of granulosa cells was examined via CCK8 assay. Granulosa cells were transfected and cultured in a 100 μL volume in 96-well plates (1 × 10^4^ cells/well) with six repetitions and then treated in gradient times (12, 24, 36 and 48 h). Then each well was added 10 μL CCK8 reagents (ZETATM life, Beijing, China) and cultivated for 3 h at 37 °C in the dark. After 20 min of shaking at room temperature, Epoch microplate reader (Biotek, Winooski, USA) was used to detect the absorbance of mixtures at 450 nm.

### EdU staining

The proliferation of granulosa cells was examined via EdU staining. Granulosa cells were transfected and cultured in a 100 μL volume in 96-well plates (1 × 10^4^ cells/well) with three repetitions and harvested after transfection for 24 h. Then cells were stained with EdU (Ribobio, Guangzhou, China) for 2 h at a final concentration of 50 μmol/L and with DAPI at room temperature for 15 min after PBS washed three times. The cells were observed by Fluorescence microscopy (IX71, Olympus, Japan).

### Flow cytometry assay (FCM)

Granulosa cells were seeded and harvested in 6-well plates when post-transfected in 24 h. Then the cell cycle of granulosa cells was measured using cell cycle staining kit (SeaBiotech, Shanghai, China) according to the manufacturer’s specifications. The apoptosis of granulosa cells were measured using Annexin V-FITC PI staining apoptosis assay kit (SeaBiotech, Shanghai, China), and the percentage of apoptotic cells were detected behind 200 μL mixtures staining with 10 μL annexin V-FITC and 5 μL propidium iodide (PI) in 30 min. All the flow cytometry assays were detected with a FACSCalibur flow cytometer (BD Biosciences).

### Animal experimental treatment

Twelve healthy SPF C57BL/6j female mice aged 7–8 weeks and weighing 22 ± 2 g were selected. The mice were provided by Xi’an Jiaotong University Medical Laboratory Animal Center (approval number: SCXK (Shaanxi) 2007-001). All animals were under protocols approved by the Institutional Animal Care and Use Committee. After 1 week of adaptive feeding, the mice were randomly divided into four groups and injected with intraperitoneal drugs on the 1^st^, 3^rd^ and 7^th^ day. The test control group was named NC (100 μL saline), whereas the test groups were named miR-101-3p agonist (miR-101-3p-ag; 10 nmol per time, with 100 μL saline), miR-101-3p antagonist (miR-101-3p-antag; 20 nmol per time, with 100 μL saline) or siRNA-STC1 (si-STC1; 20 nmol per time, with 100 μL saline) (RiboBio, Guangzhou, China). On the 9^th^ day, mice ovaries were separated and collected under sterile conditions and then fixed with 4% paraformaldehyde after washed with pre-cooled normal saline [30].

### Fluorescence *in situ* hybridisation (FISH)

After tissue fixation, dehydration, slicing and dewaxing paraffin sections to water, the sections were boiled in the repair solution for 10 min. Proteinase K (20 μg/mL) was added dropwise and digested at 37 °C for 30 min and wash with PBS for 5 min at 3 times. Then the pre-hybridisation solution was added and incubated at 37 °C for 1 h. The mmu-miR-101a probe containing a hybridisation solution was added at a concentration of 8 ng/μL, and hybridisation was performed at 37 °C overnight. A DAPI staining solution was added to the sections and incubated for 8 min in the dark, and anti-fluorescence quenching sealing tablets were mounted after washing. The nucleus stained by DAPI (Servicebio, Wuhan, China) was blue under ultraviolet excitation, and the positive expression was green fluorescence of the corresponding fluorescein-labelled FAM (488). The images were observed using a upright fluorescence microscope (Nikon, Tokyo, Japan).

### Immunohistochemistry

Control and treated ovaries were collected and fixed in 4% paraformaldehyde, incubated in a 20% sucrose solution at 4 °C overnight. Then the sections were added with 0.3% hydrogen peroxide in methanol solution and permeabilised with 0.3% Triton X100 for 30 min. The sections were rinsed three times (5 min each) in PBS at room temperature. Serum dilution (100 mL PBS with 1 g bovine serum albumin and 0.08 g sodium azide) was applied to STC1 primary antibodies (1:300, Abcam, London, UK) at 4 °C overnight. After washed in PBS for 5 min in three times, a biotin-conjugated anti-rabbit secondary antibody (1:100, Invitrogen, Carlsbad, USA) was added at room temperature for 2 h. Finally, the sections were incubated with SABC-DyLight 488 (SABC, 1:800, Boster, Shanghai, China) and DAPI (1:1000, Boster, Shanghai, China) for 30 min. SABC-positive cells were dyed in brown, while cell nuclei were dyed with DAPI in blue. Image Pro Plus image analysis software was used for quantitative immunohistochemical analysis.

### Hematoxylin-eosin (HE) staining

The dissected ovaries were fixed in 4% paraformaldehyde and either frozen in cryomatrix or embedded in paraffin. The sections were stained with Harris hematoxylin (Bioss, Beijing, China) for 7 min, washed with tap water and stained in eosin staining solution (Bioss, Beijing, China) for 1–3 min. The sections were treated with neutral balsam after drying, and observed by fluorescence microscope (Nikon, Tokyo, Japan).

### Statistical analysis

All data were analyzed by SPSS 19.0 and presented as mean ± SD of three independent experiments. Student’s ttest or one-way ANOVA followed by determination of the least significant difference (LSD) for *post-hoc* multiple comparisons was applied to compare differences of means between two or among more than two groups using GraphPad Prism 7 software. Significance levels or *P* values were stated in each corresponding figure legend. Significance was accepted at the level of *P* < 0.05 (*P < 0.05, **P < 0.01).

## Results

### Molecular analysis and aligning to sequencing data

To obtain a global scope of sequencing transcriptome, total RNA from goat granulosa cells transfected with miR-101-3p mimics and NC was used to institute RNA libraries through RNA-Seq. Prior to high throughput sequencing, molecular analysis was performed to evaluate the miR-101-3p expression levels. We acquired 72,795,015 and 73,658,977 clean reads per sample after filtering the unique adaptor sequences consisting of N and low-quality sequences. Approximately 85.313% and 85.307% total mapped reads were acquired from the reference genome (Table 1). In the miR-101-3p group, 3.612% or 81.656% was mapped either to multiple or to unique genomic locations, whereas the NC group exhibited 3.135% or 82.171% reads. The distribution of effective sequences in the reference genomes was measured using the standard metrics of exon, intron and intergenic reads. Table S2 shows the individual distributions. Before performing differential expression analysis, the correlation among the repeated samples was examined. The heat map exhibited that R^2^ values among three replicates originating from the same treatment were greater than 0.95 (Fig. S2a), showing the slight differences among samples in each treatment.

**Table 1:** Summary of sequence read alignments to the reference genome

Category	miR-101-3p		Negative control (NC)	
	Reads number	Percentage	Reads number	Percentage
Total reads	72,795,015	100%	73,658,977	100%
Total mapped reads	62,103,473	85.313%	62,835,877	85.307%
Multiple mapped reads	2,660,677	3.655%	2,309,196	3.135%
Uniquely mapped reads	59,442,796	81.656%	60,526,526	82.171%

Total reads: total number of sequencing reads. Total mapped reads: the reads that can aligned to reference sequence and the ratio of it. Multiple mapped reads: in total mapped reads, reads aligned to two or more places. Uniquely mapped reads: in total mapped reads, reads aligned to only one postion

### Analysis of DEGs after miR-101-3p overexpression

Next we examined the differentially expressed unigenes after miR-101-3p overexpression to determine the underlying mechanisms mediated by miR-101-3p. As multiple DEGs were present between two libraries, genes coinciding with the specified criterion of |log_2_FoldChange| > 1 and *P*-values < 0.05 were centralised. We identified 142 DEGs compared miR-101-3p with NC libraries, including 78 down-regulated and 64 up-regulated genes (Fig. S2b). Noticeably, in the miR-101-3p group, the five down-regulated DEGs that expressed most significant were *NDUFA4L2* (− 1.24769817-fold, *P* = 8.87E-50), *C4BPA* (− 1.490250616-fold, *P* = 3.95E-29), *STC1* (− 1.160429719-fold, *P* = 4.25E-27), *LOC102189835* (− 1.033859332-fold, *P* = 1.10E-12) and *MCOLN3* (− 1.474046641-fold, *P* = 1.05E-5) as well as up-regulated DEGs were *FSHB* (1.358101058-fold, *P* = 4.42E-13), *BMPER* (1.375372372-fold, *P* = 3.28E-6), *ADAMTS15* (2.00772808-fold, *P* = 9.94E-6), *LOC102185049* (1.101564295-fold, *P* = 2.32E-5) and *LOC102178901* (2.699102983-fold, *P* = 3.59E-5) compared with NC. The five down-regulated DEGs with highest fold change were *LOC108633912* (inf-fold, *P* = 0.001479515), *LOC102187646* (inf-fold, *P* = 0.031226999), *C3H1orf168* (inf-fold, *P* = 0.023013464), *EDN2* (− 4.13414302-fold, *P* = 0.035939657) and *RETN* (− 3.545297992-fold, *P* = 0.021438006) as well as the five up-regulated DEGs were *FAM159A* (inf-fold, *P* = 0.020739771), *KRT14* (inf-fold, *P* = 0.012713064), *LOC106501921* (inf-fold, *P* = 0.023789854), *SEZ6* (inf-fold, *P* = 0.024290615) and *KNDC1* (inf-fold, *P* = 0.03442371). All up- and down-regulated DEGs identified are presented in Table S4.

### Functional classification analysis of DEGs

To further explore the physiological processes associated with DEGs, we implemented GO analysis by operating queries for the respective DEG against the GO database, which furnishes information correlated with three independent ontology categories: cellular component, molecular function and biological process [28]. Fig. S2c and Table S5 show the results of the GO analysis of DEGs. Overall, the DEGs were categorised into 712 functional groups and subsequently into three ontologies, that is, 70 (9.8%), 569 (79.9%) and 73 (10.3%) terms in accordance with molecular functions, biological processes and cellular components, respectively. Binding (GO:0005488, 23 out of 56 genes), single-multicellular organism process (GO:0044707, 30 out of 56 genes) and cell part (GO:0044464, 39 out of 56 genes) were mostly dominant terms in each primary category.

Diverse genes generally cooperate with one another to perform their biological capabilities. KEGG is an effective pathway-related database and pathway enrichment analysis of enriched metabolic or signal transduction pathways of DEGs [29]. Among those genes with KEGG pathway annotation, about 84.5% DEGs (120/142) were identified in 68 enriched pathways (Fig. S2d and Table S6). The pathway term that exhibited the highest level of significance was neuroactive ligand-receptor interaction (KO:04080, *P* = 0.006076663) with seven DEGs. Natural killer cell-mediated cytotoxicity (KO:04650, *P* = 0.005498505) and cytokine-cytokine receptor interaction (KO:04060, *P* = 0.05040407) were also among the significantly enriched pathways with five DEGs each. Complement and coagulation cascades (KO:04610, *P* = 0.003356877) with four DEGs, natural killer cell-mediated cytotoxicity (KO:04650, *P* = 0.005498505) with five DEGs and neuroactive ligand-receptor interaction (KO:04080, *P* = 0.006076663) with seven DEGs were the three most notably enriched pathways.

### Verification of differential gene expression through RT-qPCR

**Fig. 1:**
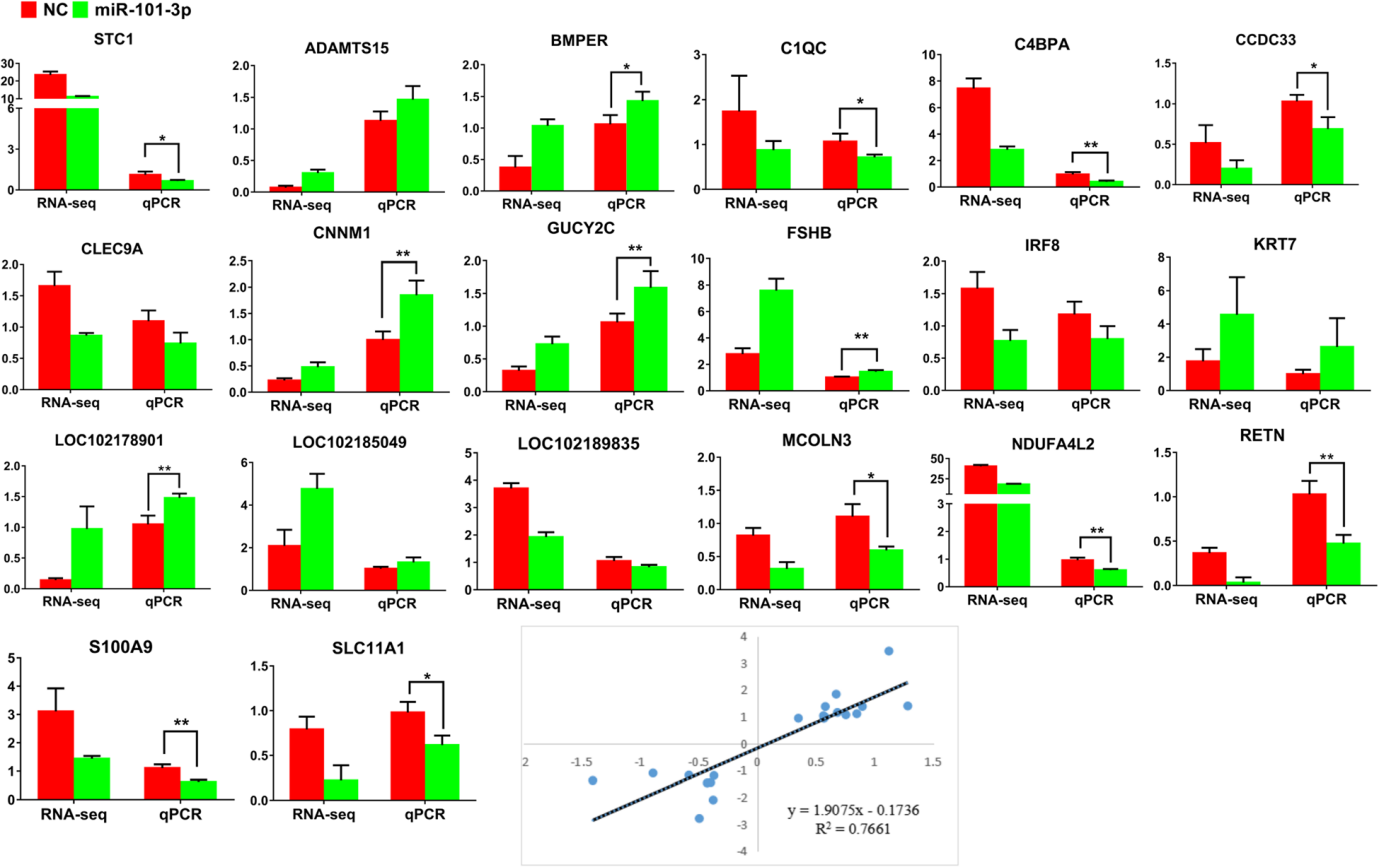
Verification of differential genes expressions. RT-qPCR quantifies 12 down-regulated DEGs (*STC1, NDUFA4L2, C4BPA, LOC102189835, CLEC9A, MCOLN3, RETN, SLC11A1, C1QC, IRF8, S100A9* and *CCDC33*) and 8 up-regulated DEGs (*FSHB, KRT7, LOC102185049, CNNM1, GUCY2C, BMPER, ADAMTS15* and *LOC102178901*) in goat granulosa cells, which are transfected with miR-101-3p mimics or negative control (NC) for 24 h. β-actin is used as an internal control. The linear regression equation indicates the correlation coefficient R^2^ value between genes. Values are expressed as mean ± SD of *n* = 3. * *P* < 0.05, ** *P* < 0.01

We selected 12 down-regulated (*STC1, NDUFA4L2, C4BPA, LOC102189835, CLEC9A, MCOLN3, RETN, SLC11A1, C1QC, IRF8, S100A9* and *CCDC33*) and 8 up-regulated (*FSHB, KRT7, LOC102185049, CNNM1, GUCY2C, BMPER, ADAMTS15* and *LOC102178901*) genes to verify the expression profiles acquired through RNA-Seq. The RT-qPCR results showed that compared with NC, miR-101-3p inhibited *STC1, NDUFA4L2, C4BPA, MCOLN3, RETN, SLC11A1, C1QC, S100A9* and *CCDC33* as well as promoted *FSHB, CNNM1, GUCY2C, BMPER* and *LOC102178901* in mRNA levels significantly. Although the mRNA differences of *LOC102189835, CLEC9A, IRF8, ADAMTS15, KRT7* and *LOC102185049* weren’t prominent, they were still consistent with the sequencing detections (Fig. 1). The linear regression method proved that the correlation coefficient R^2^ = 0.77 (Fig. 1). Hence, RNA-Seq can yield appropriate data for mRNA distinct expression analysis after miR-101-3p overexpression in granulosa cells.

### MiR-101-3p specifically targets *STC1*

**Fig. 2:**
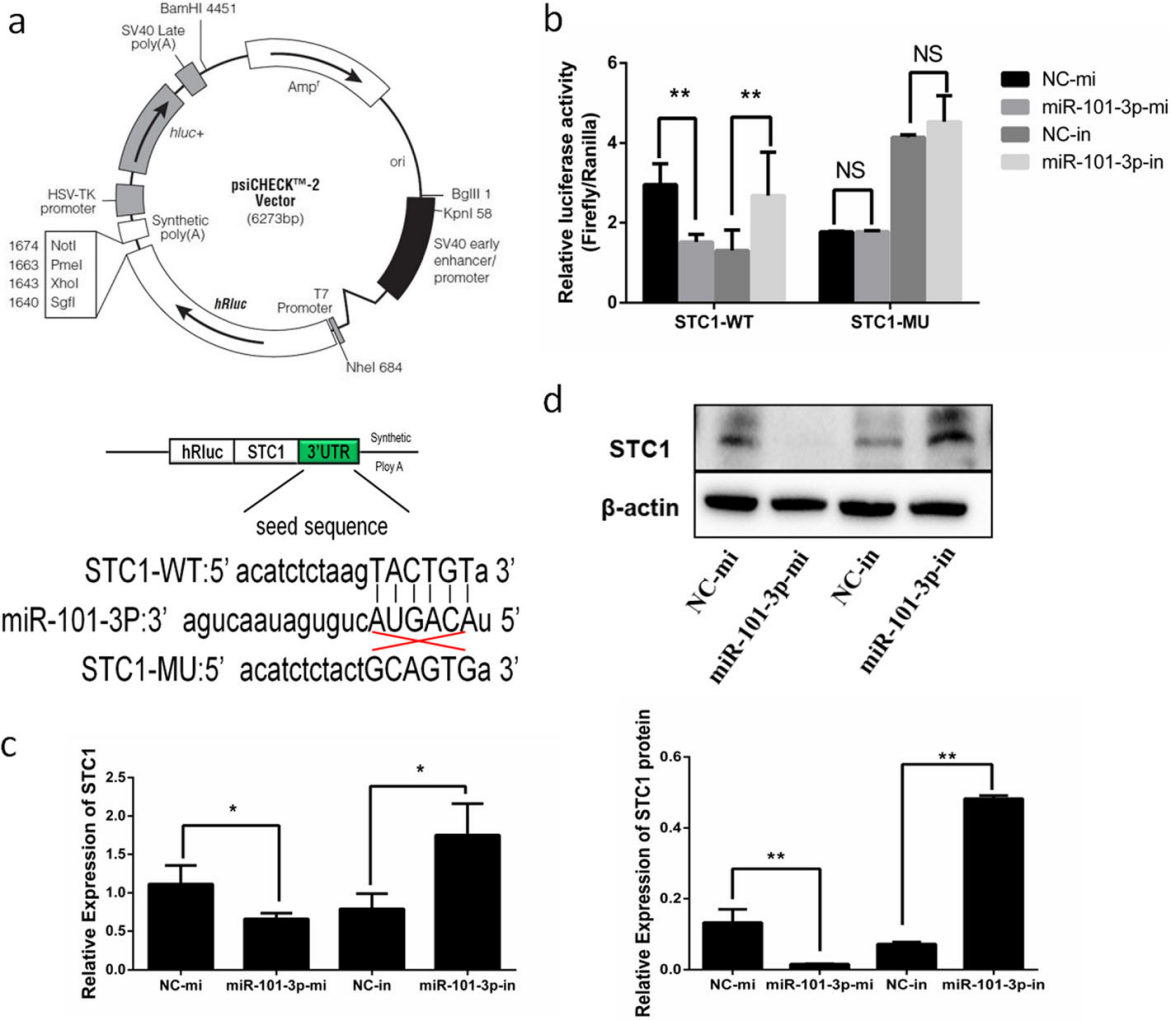
MiR-101-3p specifically targets *STC1* in goat granulosa cells. **a** Target sites for miR-101-3p in the *STC1* 3′-UTR and the construction of the luciferase expression vector (Luc) fused with the *STC1* 3′-UTR. wt represents the Luc reporter vector with the wild-type STC1 3′-UTR; mu represents the Luc reporter vector with the mutation at the miR-101-3p site in *STC1* 3′-UTR. **b** After HEK293T cells transfected miR-101-3p mimics (miR-101-3p-mi) or mimics NC (NC-mi), miR-101-3p inhibitors (miR-101-3p-in) or inhibitors NC (NC-in) with wt-/mu-Luc reporter vectors for 48 h, the relative luciferase activities are measured. The (**c)** mRNA or **(d)** protein expressions of *STC1* in granulosa cells transfected with miR-101-3p-mi/-in or NC-mi/-in for 24 h or 48 h are quantified using RT-qPCR or Western blot, respectively. β-actin is used as an internal control. Values are expressed as mean ± SD of *n* = 3. * *P* < 0.05, ** *P* < 0.01. NS means no significance

Based on a target prediction algorithm (TargetScan, http://www.targetscan.org/), we probed into the novel underlying molecular targets of miR-101-3p. Among these potential targets, *STC1* attracted our attention because it was not only a key regulator of diverse metabolic processes [22–26], but also a DEG in terms of RNA-Seq approach. As miRNA functions and suppresses the expression of target genes by binding to 3′-UTR, we implemented a two-tier luciferase assay in HEK293T cells using the psiCHECK-2 vector and framed luciferase reporters with the wt- or mu-STC1-3′-UTR (Fig. 2a), respectively. Figure 2b shows that compared with control, miR-101-3p overexpression prominently inhibited relative luciferase activities in cells transfected with psiCHECK2-wt-STC1 but not in the mutant plasmid. The opposite results were found in miR-101-3p-in-treated cells (Fig. 2b).

To affirm whether *STC1* expression is regulated by miR-101-3p, we detected its mRNA and protein levels in granulosa cells using RT-qPCR and Western blot respectively. We observed significant *STC1* mRNA decrease in miR-101-3p-mi group and increase in miR-101-3p-in group compared with controls (Fig. 2c). The changes in the protein expression of STC1 showed the same results (Fig. 2d), revealing that miR-101-3p targets the 3′-UTR of *STC1* specifically and functions as a demotivated mediator.

### MiR-101-3p promotes steroid hormone synthesis via *STC1*

**Fig. 3:**
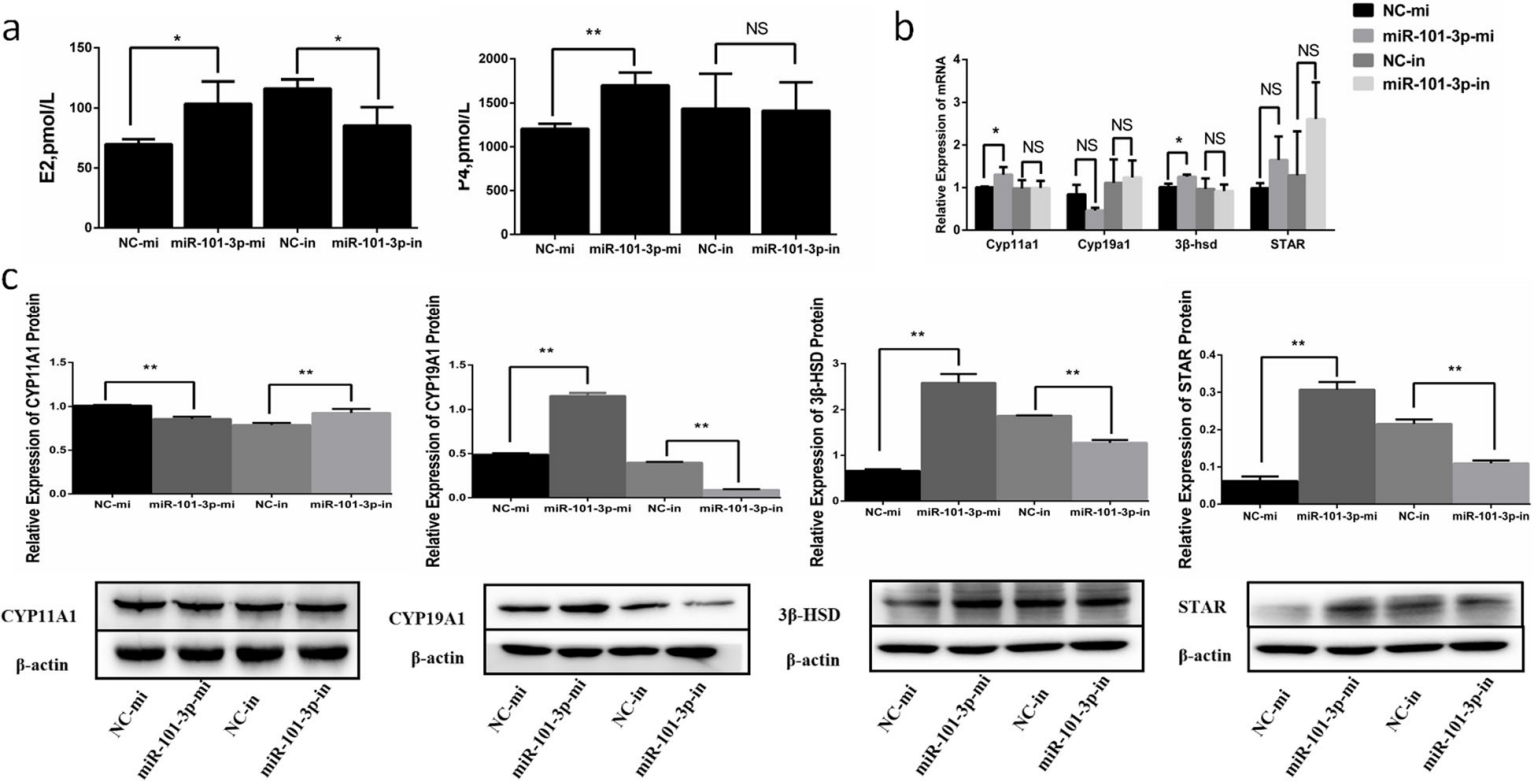
MiR-101-3p promotes steroid hormone synthesis. Goat granulosa cells are transfected with miR-101-3p mimics (miR-101-3p-mi) or mimics NC (NC-mi), miR-101-3p inhibitors (miR-101-3p-in) or inhibitors NC (NC-in). **a** After 24 h, cell-free supernatants are collected and estrogen (E2) and progesterone (P4) secretions are measured in 50 μL supernatants using ELISA kits. The **(b)** mRNA or **(c)** protein expressions of steroid hormone synthesis-associated vital genes *CYP11A1, CYP19A1, 3β-HSD* and *STAR* are quantified using RT-qPCR or Western blot after 24 or 48 h treatment, respectively. β-actin is used as an internal control. Values are expressed as mean ± SD of *n* = 3. * *P* < 0.05, ** *P* < 0.01. Superscripts (a, b, c) show significant difference. NS means no significance

**Fig. 4:**
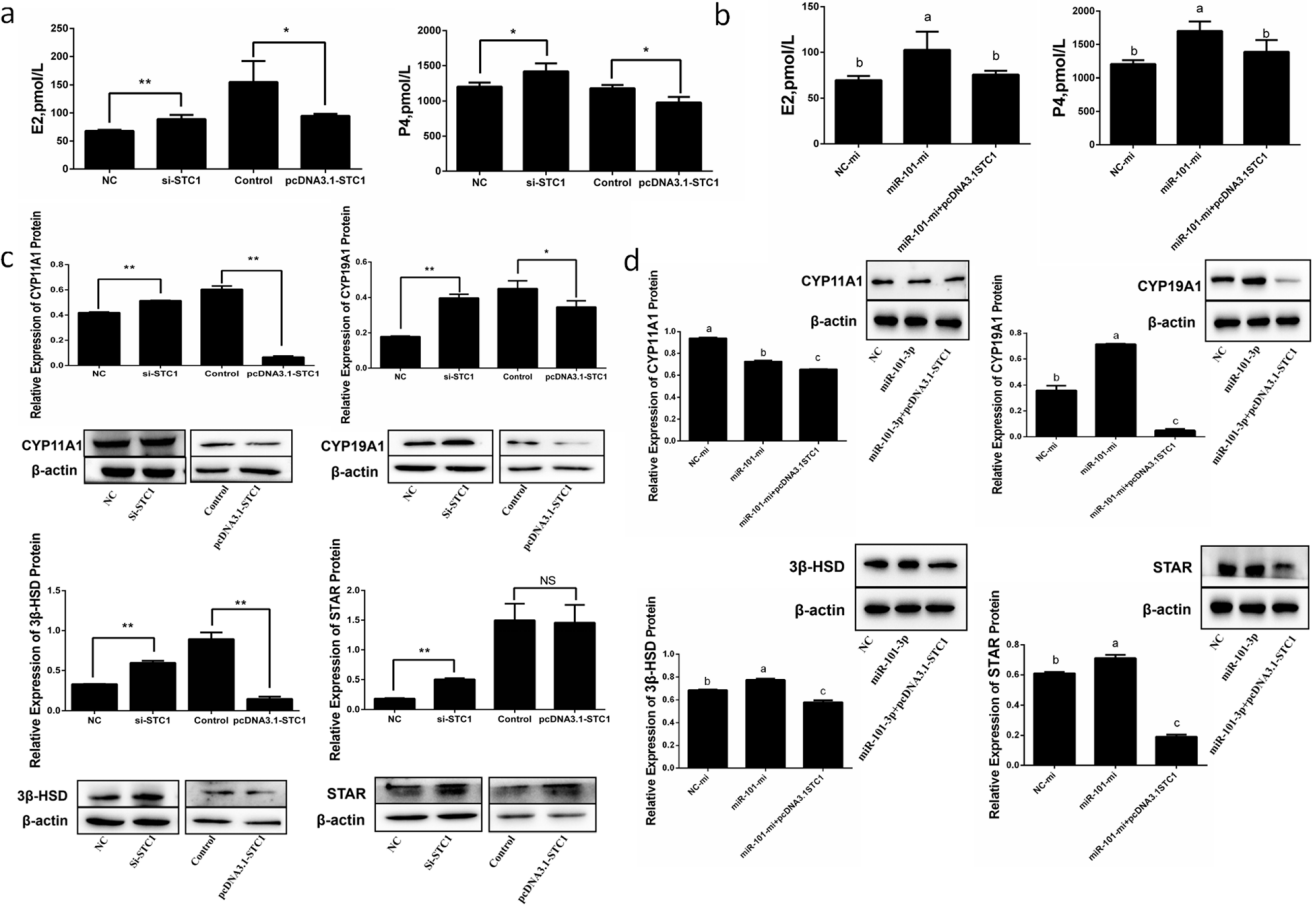
MiR-101-3p promotes steroid hormone synthesis via *STC1*. ELISA kits measure oestrogen (E2) and progesterone (P4) secretions in 50 μL cell-free supernatants in goat granulosa cells transfected with **(a)** siRNA-STC1 (si-STC1) or NC, pcDNA3.1-STC1 or pcDNA3.1 (control) or **(b)** miR-101-3p mimics (miR-101-3p-mi), mimics NC (NC-mi) or co-transfected miR-101-3p-mi with pcDNA3.1-STC1 for 24 h. The protein expressions of steroid hormone synthesis-associated vital genes STAR, CYP11A1, CYP19A1 and 3β-HSD are quantified using Western blot in goat granulosa cells transfected with **(c)** si-STC1 or NC, pcDNA3.1-STC1 or pcDNA3.1 (control) or **(d)** miR-101-3p-mi, NC-mi or co-transfected miR-101-3p-mi with pcDNA3.1-STC1 after 48 h transfection. β-actin is used as an internal control. Values are expressed as mean ± SD of *n* = 3. * *P* < 0.05, ** *P* < 0.01. Superscripts (a, b, c) show significant difference. NS means no significance

After transfection of miR-101-3p mimics or inhibitors in granulosa cells for 24 h, the levels of E2 and P4 in the cell-free supernatants were detected by ELISA to investigate the effects of miR-101-p on steroid hormone synthesis. Cells with miR-101-3p overexpression showed enhanced E2 and P4 secretions (Fig. 3a). MiR-101-3p inhibition decreased E2 production but had no significant effects on P4 (Fig. 3a). Subsequently, we probed into whether miR-101-3p functions on steroid hormone synthesis of granulosa cells by inhibiting *STC1*. A vector containing *STC1* coding sequence (CDS) or si-STC1 was transfected into granulosa cells for 24 h. *STC1* overexpression blocked E2 and P4 secretions, which were improved in si-STC1-treated cells (Fig. 4a). Meanwhile, *STC1* significantly reduced E2 and P4 secretions when miR-101-3p was overexpressed (Fig. 4b).

E2 and P4, as vital steroid hormones, their synthesis depends on a series of enzymatic transformation processes [31]. In order to further study the regulation of miR-101-3p in steroid hormones synthesis, RT-qPCR and Western blot were used to detect the expressions of steroid hormone synthesis-associated vital genes (*CYP11A1, CYP19A1, 3β-HSD* and *STAR*) in granulosa cells. At mRNA levels, miR-101-3p elevation increased *CYP11A1* and *3β-HSD* expression levels but not *CYP19A1* and *STAR* (Fig. 3b). However, no remarkable effects were observed between miR-101-3p-in and NC-in groups (Fig. 3b). At protein levels, CYP19A1, 3β-HSD and STAR expressions increased, whereas CYP11A1 expression decreased in miR-101-3p-mi group (Fig. 3c). MiR-101-3p depletion reduced the levels of CYP19A1, 3β-HSD and STAR as well as increased CYP11A1 expression significantly (Fig. 3c). Decline of *STC1* promoted the protein levels of CYP11A1, CYP19A1, 3β-HSD and STAR. On the contrary, suppressive expressions of CYP11A1, CYP19A1 and 3β-HSD in pcDNA3.1-STC1 group were observed (Fig. 4c). Co-expression of miR-101-3p with *STC1* exhibited an adiaphorous effect on CYP19A1, 3β-HSD and STAR but not on CYP11A1 (Fig. 4d). These findings demonstrate that miR-101-3p promotes E2 and P4 secretions in granulosa cells via *STC1* by regulating *CYP11A1, CYP19A1, 3β-HSD* and *STAR* steroid hormone synthesis-associated genes.

### MiR-101-3p inhibits granulosa cell proliferation via *STC1*

**Fig. 5:**
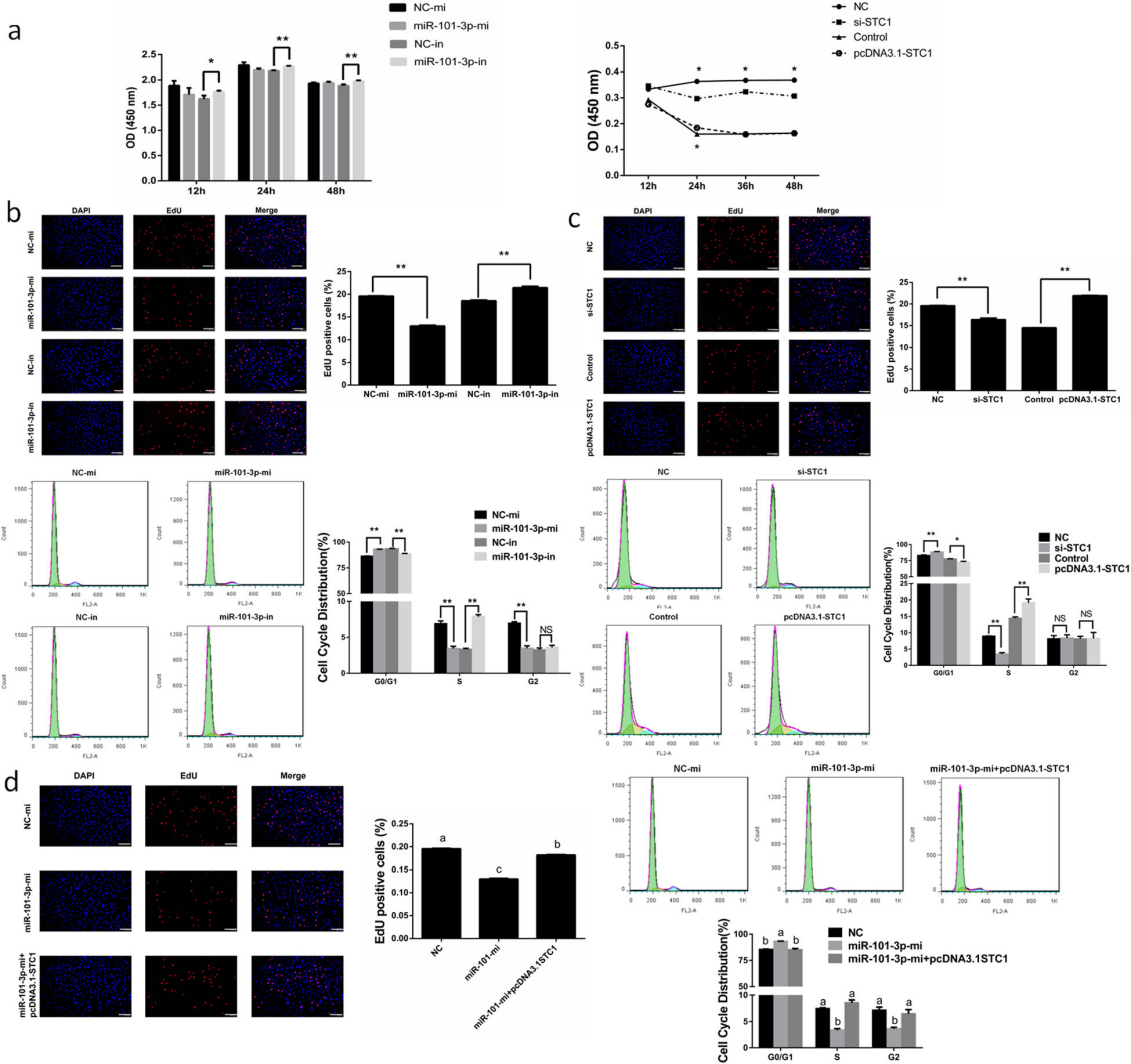
MiR-101-3p inhibits granulosa cell proliferation via *STC1*. Goat granulosa cells are transfected with miR-101-3p mimics (miR-101-3p-mi) or mimics NC (NC-mi), miR-101-3p inhibitors (miR-101-3p-in) or inhibitors NC (NC-in), siRNA-STC1 (si-STC1) or NC, pcDNA3.1-STC1 or pcDNA3.1 (control). **a** Cell viability is standardized by relative absorbance (OD values at 450 nm) and determined using CCK8 assay at 12, 24, 48 and 72 h time points. After 24 h transfection, **(b**, **c)** granulosa cells in the S-phase are stained with EdU in red, whereas cell nuclei are dyed with DAPI in blue. The ratio of red and blue dyed cell numbers represents the percentage of EdU positive cells. Cell cycle distribution of granulosa cells is measured using flow cytometry. **d** The percentage of EdU positive cells or cell cycle distribution is detected using above-mentioned methods in granulosa cells transfected with miR-101-3p-mi or NC-mi or co-transfected miR-101-3p-mi with pcDNA3.1-STC1 for 24 h. Values are expressed as mean ± SD of *n* = 3. * *P* < 0.05, ** *P* < 0.01. Superscripts (a, b, c) show significant difference. NS means no significance

CCK8 and EdU analyses were performed for the detection of cell viability and proliferation following cell transfected with miR-101-3p mimics or inhibitors. Granulosa cell viability was standardized by relative absorbance (OD values at 450 nm). The results showed a slight decrease in relative absorbance after miR-101-3p overexpression. By contrast, miR-101-3p inhibition significantly increased cell viability compared with control in 12, 24 and 48 h (Fig. 5a). These results were similar with those of the EdU staining assay, showing decrease or increase in the number of S-phage cells after treatment with miR-101-3p mimics or inhibitors (Fig. 5b). The influence of miR-101-3p on granulosa cell cycle was detected using flow cytometry. Granulosa cells after miR-101-3p overexpression showed 93.11% cells at G0/G1 phase, 3.43% cells at S phase and 3.46% cells at G2 phase compared to 83.15%, 6.89% and 6.96% cells in NC group (Fig. 5b). We observed reductive or incremental cell numbers at G0/G1 (88.51%) or S phase (7.88%), but with no prominent effects on cells at G2 phase (3.61%) in miR-101-3p-in group compared with the percentages in NC-in group (93.41%, 3.32% and 3.27%) (Fig. 5b). Thus, we conclude that miR-101-3p inhibits the proliferation of goat granulosa cells. After treatment in 12, 24, 36 and 48 h, granulosa cell viability was notably inhibited in si-STC1 group compared with NC. The cell viability was elevated in 24 h between pcDNA3.1-STC1 and control groups (Fig. 5a). Cells transfected with si-STC1 or pcDNA3.1-STC1 vectors decreased or increased the number of S-phage cells (Fig. 5c). *STC1* also attenuated miR-101-3p-weakened effects on cell proliferation by increasing EdU positive cells (Fig. 5d). Silencing *STC1* promoted cells to G0/G1 phase (88.15%) and reduced cell numbers at S phase (3.47%) compared to 82.90% and 8.94% cells in NC group (Fig. 5c). The number of granulosa cells was repressed at G0/G1 phase (72.57%) but elevated at S phase (19.16%) after *STC1* overexpression compared with the percentages in control group (77.49% and 14.40%) (Fig. 5c). Co-transfection group also showed a neutral result regarding cell cycle distribution, 84.97% cells at G0/G1 phase, 8.57% cells at S phase and 6.46% cells at G2 phase compared to 93.01%, 3.36% and 3.63% cells in miR-101-3p-mi group (Fig. 5d). Thus, we speculate that *STC1* promotes granulosa cell proliferation and miR-101-3p can inhibit granulosa cell proliferation via *STC1*.

**Fig. 6:**
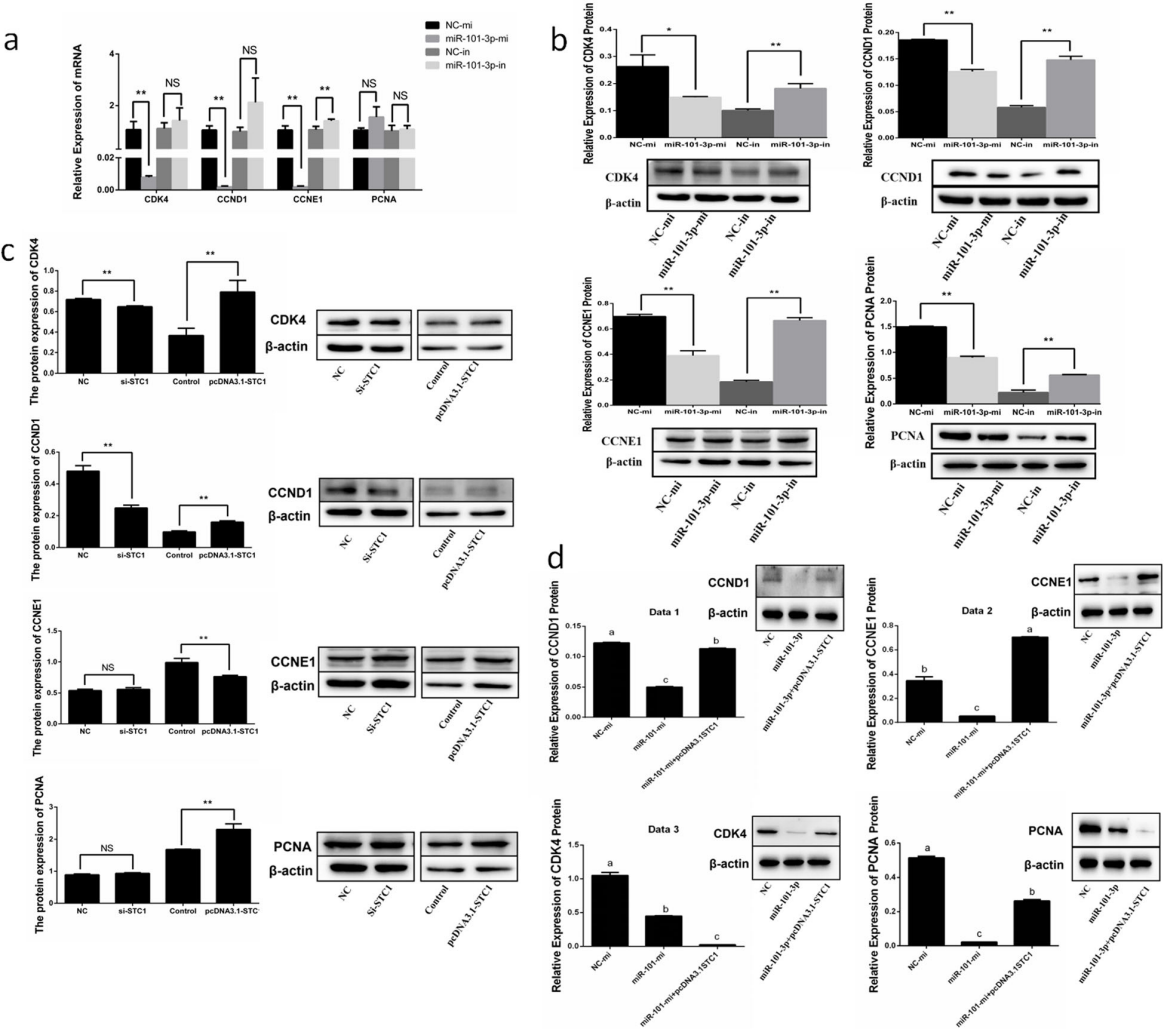
MiR-101-3p inhibits the expressions of cell proliferation-related genes via *STC1*. Goat granulosa cells are transfected with miR-101-3p mimics (miR-101-3p-mi) or mimics NC (NC-mi), miR-101-3p inhibitors (miR-101-3p-in) or inhibitors NC (NC-in). The **(a)** mRNA or **(b)** protein expressions of cell proliferation-related genes (*CDK4, CCND1, CCNE1* and *PCNA*) are detected using RT-qPCR or Western blot after 24 h or 48 h transfection. Western blot measures the protein expressions of CDK4, CCND1, CCNE1 and PCNA in granulosa cells transfected with **(c)** siRNA-STC1 (si-STC1) or NC, pcDNA3.1-STC1 or pcDNA3.1 (control) or **(d)** miR-101-3p-mi or NC-mi or co-transfected miR-101-3p-mi with pcDNA3.1-STC1 for 48 h. β-actin is used as an internal control. Values are expressed as mean ± SD of *n* = 3. * *P* < 0.05, ** *P* < 0.01. Superscripts (a, b, c) show significant difference. NS means no significance

To further explore the regulatory mechanism of miR-101-3p’s effects on granulosa cell proliferation, we detected the expressions of cell proliferation-related genes (*CDK4, CCND1, CCNE1* and *PCNA*). Figure 6a shows that miR-101-3p overexpression suppressed the mRNA levels of *CDK4, CCND1* and *CCNE1*, whereas miR-101-3p inhibition facilitated the mRNA levels of *CCNE1*. Down-regulation of CDK4, CCND1, CCNE1 and PCNA protein expression was observed in miR-101-3p-mi group, whereas opposite results were found in miR-101-3p-in group (Fig. 6b). Cells transfected with si-STC1 blocked the expression of CDK4 and CCND1 but showed slight effects on CCNE1 or PTEN (Fig. 6c). *STC1* overexpression accelerated CKD4, CCND1 and PCNA but restrained CCNE1 protein levels (Fig. 6c). Co-expression of miR-101-3p with *STC1* exhibited an adiaphorous effect on CCND1, CCNE1 and PCNA but not on CDK4 (Fig. 6d). These findings indicate that miR-101-3p is capable of inhibiting granulosa cell proliferation by effects on *CDK4, CCND1, CCNE1* and *PCNA* proliferation-related genes via *STC1*.

### MiR-101-3p promotes granulosa cell apoptosis via *STC1*

**Fig. 7:**
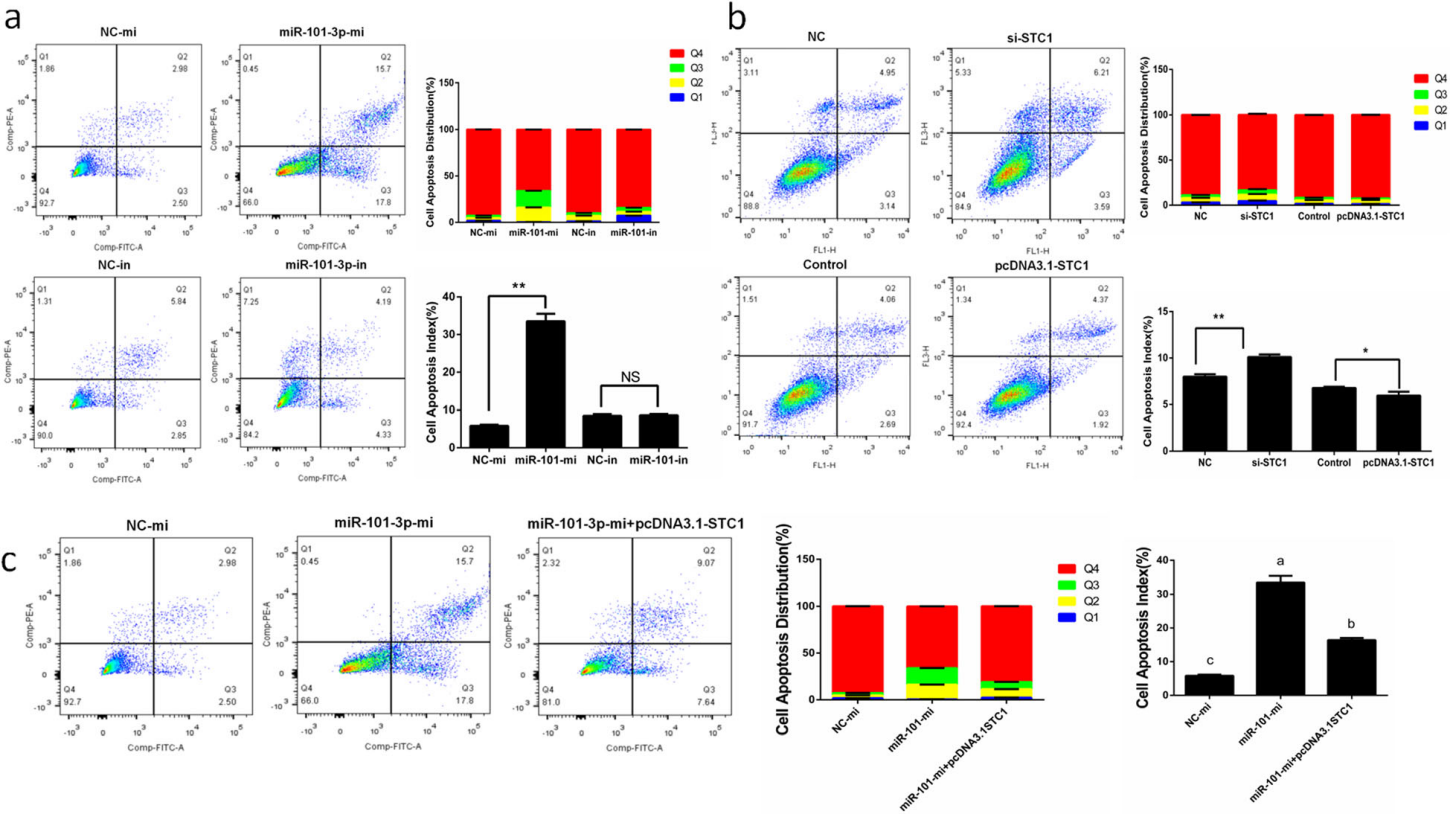
MiR-101-3p promotes granulosa cell apoptosis via *STC1*. Goat granulosa cells are transfected with **(a)** miR-101-3p mimics (miR-101-3p-mi) or mimics NC (NC-mi), miR-101-3p inhibitors (miR-101-3p-in) or inhibitors NC (NC-in), **(b)** siRNA-STC1 (si-STC1) or NC, pcDNA3.1-STC1 or pcDNA3.1 (control), **(c)** miR-101-3p-mi or NC-mi or co-transfected miR-101-3p-mi with pcDNA3.1-STC1 for 24 h. The apoptotic rates of granulosa cells are detected by flow cytometry. Histograms show the apoptotic cell distribution and percentage. Q1 means death cells, Q2 means late-state apoptotic cells, Q3 means early-state apoptotic cells and Q4 means viable non-apoptotic cells. Values are expressed as mean ± SD of *n* = 3. * *P* < 0.05, ** *P* < 0.01. Superscripts (a, b, c) show significant difference. NS means no significance

**Fig. 8:**
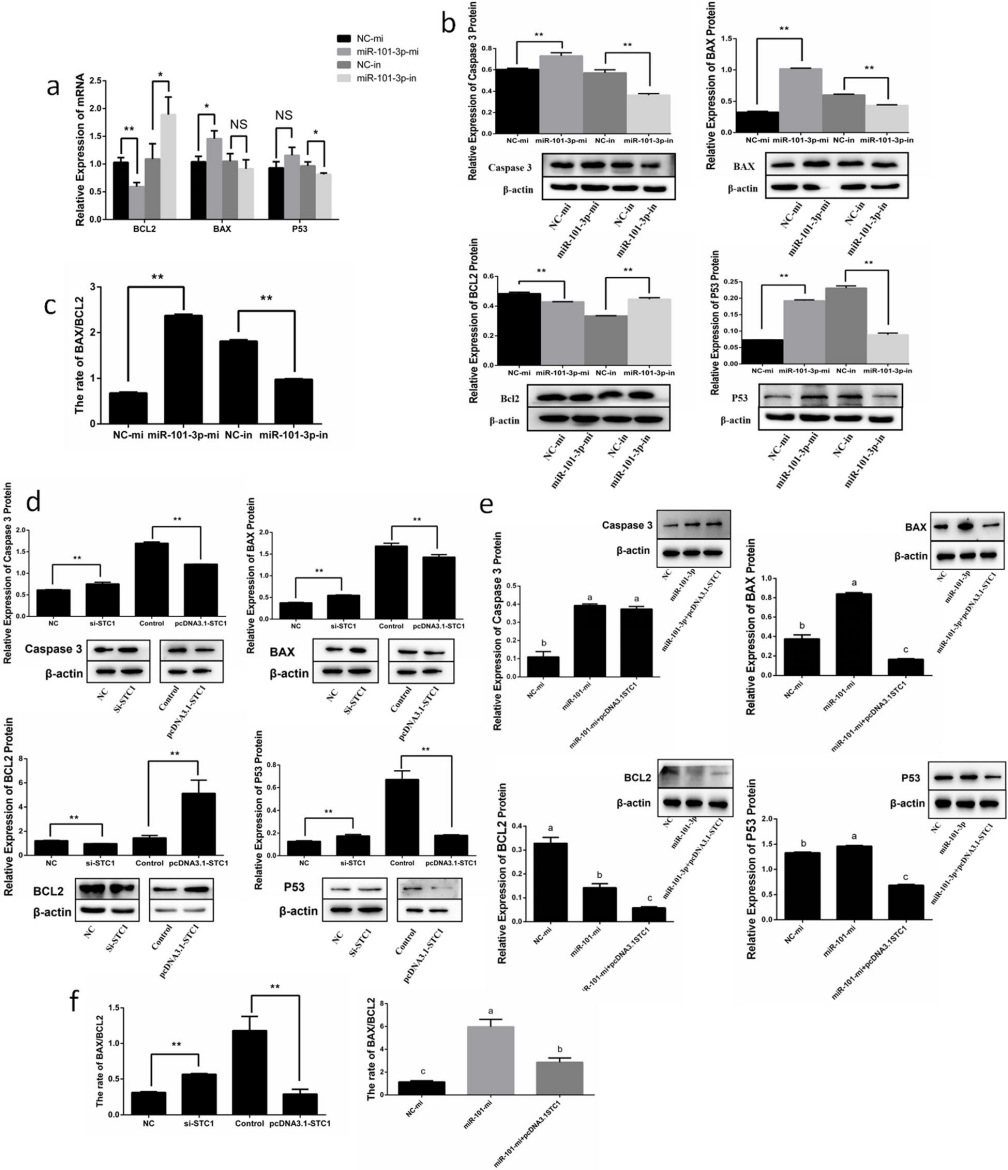
MiR-101-3p regulates the apoptotic genes in granulosa cells via *STC1*. Goat granulosa cells are transfected with miR-101-3p mimics (miR-101-3p-mi) or mimics NC (NC-mi), miR-101-3p inhibitors (miR-101-3p-in) or inhibitors NC (NC-in). **a** The mRNA expressions of apoptotic genes (*Bcl-2, Bax* and *p53*) are detected using RT-qPCR after 24 h transfection. **b** The protein expressions of apoptotic genes (Caspase 3, Bcl-2, Bax and p53) are detected using Western blot after 48 h transfection. Caspase 3, Bcl-2, Bax and p53 protein levels are detected in granulosa cells transfected with **(d)** siRNA-STC1 (si-STC1) or NC, pcDNA3.1-STC1 or pcDNA3.1 (control), **(e)** miR-101-3p-mi or NC-mi or co-transfected miR-101-3p-mi with pcDNA3.1-STC1 for 48 h using Western blot. (c, f) Histograms show the ratio of Bcl-2/Bax. β-actin is used as an internal control. Values are expressed as mean ± SD of *n* = 3. * *P* < 0.05, ** *P* < 0.01. Superscripts (a, b, c) show significant difference. NS means no significance

Flow cytometry showed that after 24 h transfection, the total apoptotic rates of granulosa cells increased in miR-101-3p-mi group (Fig. 7a). Cells treated with miR-101-3p-in took no effects on total apoptotic rates of granulosa cells, but exhibited decreased numbers of early-state apoptotic cells and increased numbers of late-state apoptotic cells (Fig. 7a). We detected the expressions of valid apoptotic genes *Bcl-2, Bax* and *p53*. MiR-101-3p elevation promoted Bax and inhibited Bcl-2 in mRNA and protein levels, and only promoted p53 protein levels (Fig. 8a, b). Cells with miR-101-3p inhibitors suppressed Bax and p53 and stimulated Bcl-2 in mRNA and protein levels (Fig. 8a, b). Furthermore, miR-101-3p decreased the ratio of Bcl-2/Bax (Fig. 8c), clarifying that miR-101-3p promotes granulosa cell apoptosis. We also detected the influence of *STC1* in granulosa cell apoptosis. Figure 7b illustrates increased total apoptotic cells after cells transfected with si-STC1. Inducing *STC1* resulted in decreased apoptotic cell numbers, and inhibited the positive effects of miR-101-3p on granulosa cell apoptosis (Fig. 7b, c). Western blot demonstrated that the protein expressions of Bax, p53 and Caspase3 were improved, accompanied by restraining Bcl-2 in si-STC1 group, whereas STC1 overexpression showed opposite results (Fig. 8d). The ratio of Bcl-2/Bax was significantly increased by *STC1* (Fig. 8f), implying that *STC1* inhibits granulosa cell apoptosis. The effects of miR-101-3p on Bax, Bcl-2 and p53 were partially decreased after *STC1* overexpression (Fig. 8e, f). Thereby it suggests that miR-101-3p promotes granulosa cell apoptosis, at least in some degree, through *STC1*.

### MiR-101-3p inhibits PI3K-AKT-mTOR pathway via *STC1*

**Fig. 9:**
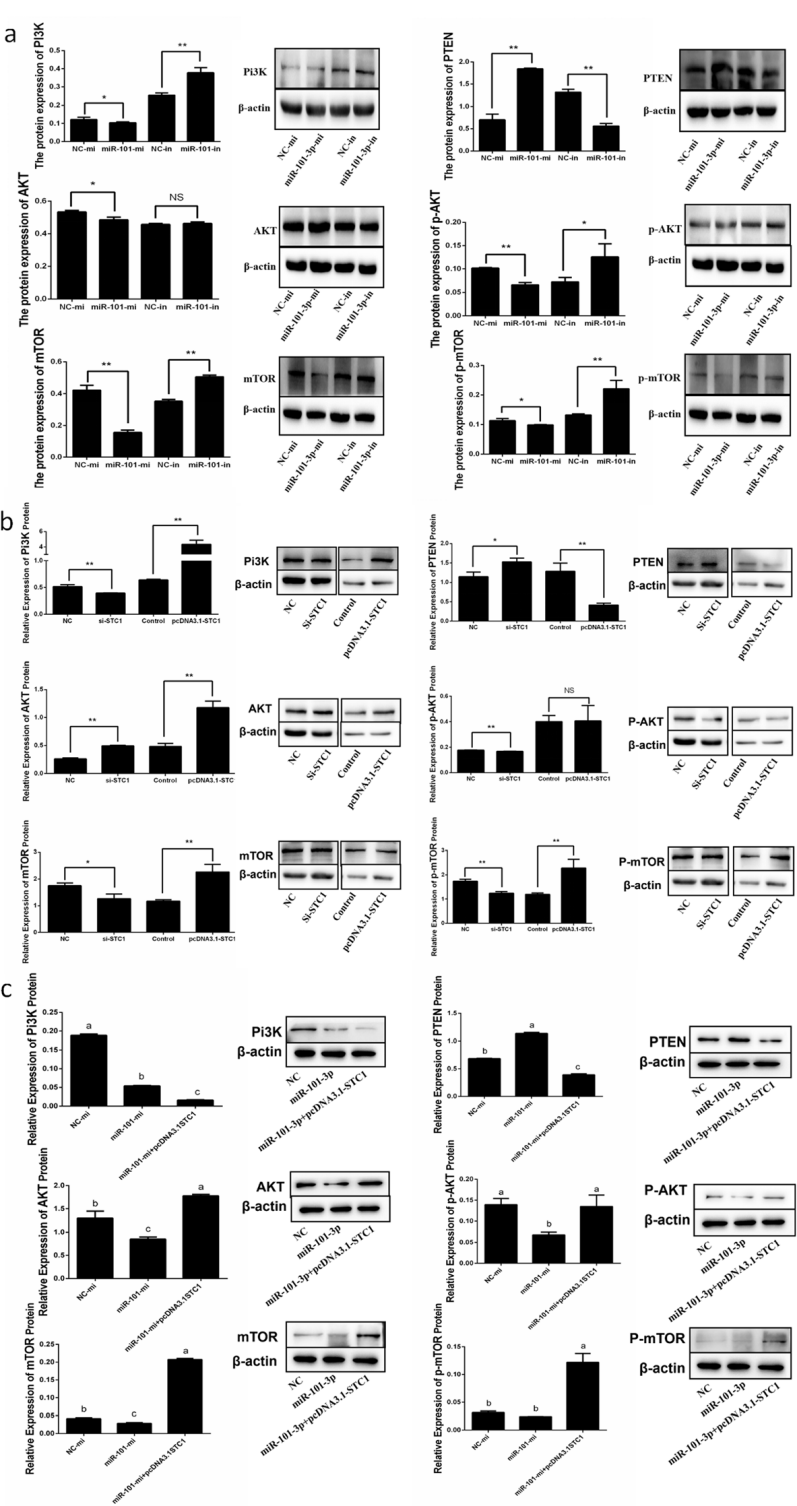
MiR-101-3p inhibits PI3K-AKT-mTOR pathway via *STC1*. Goat granulosa cells are transfected **(a)** miR-101-3p mimics (miR-101-3p-mi) or mimics NC (NC-mi), miR-101-3p inhibitors (miR-101-3p-in) or inhibitors NC (NC-in), **(b)** siRNA-STC1 (si-STC1) or NC, pcDNA3.1-STC1 or pcDNA3.1 (control), **(c)** miR-101-3p-mi or NC-mi or co-transfected miR-101–3p-mi with pcDNA3.1-STC1 for 48 h. Cytosolic protein and related phosphorylation levels for PI3K, AKT, mTOR and PTEN are analysed using Western blot. β-actin is used as an internal control. Values are expressed as mean ± SD of *n* = 3. * *P* < 0.05, ** *P* < 0.01. Superscripts (a, b, c) show significant difference. NS means no significance

PI3K-AKT-mTOR pathway plays a crucial function on cell growth, proliferation, apoptosis and other processes [32]. Therefore, we explored whether miR-101-3p and *STC1* can affect the key genes in PI3K-AKT-mTOR pathway. MiR-101-3p overexpression promoted PTEN, inhibited PI3K, AKT and mTOR protein levels, as well as triggered the activation of AKT and mTOR (Fig. 9a). MiR-101-3p inhibition significantly reduced PTEN expression and promoted PI3K and mTOR protein levels (Fig. 9a). Phosphorylation expressions of AKT and mTOR were also promoted between miR-101-3p-in and NC-in groups (Fig. 9a). These data support the passive role of miR-101-3p on PI3K-AKT-mTOR pathway. *STC1* depletion decreased the protein levels of PI3K, p-AKT, mTOR and p-mTOR but increased those of PTEN and AKT (Fig. 9b). We observed increased PI3K, AKT, mTOR and p-mTOR expressions and decreased PTEN expression after *STC1* was induced (Fig. 9b). The effects of miR-101-3p on PI3K, PTEN, AKT, p-AKT, mTOR and p-mTOR were partially neutralized after *STC1* overexpression (Fig. 9c), suggesting that miR-101-3p inhibits PI3K-AKT-mTOR pathway via *STC1*.

### Effects of miR-101-3p and *STC1* on ovarian development in mice

**Fig. 10:**
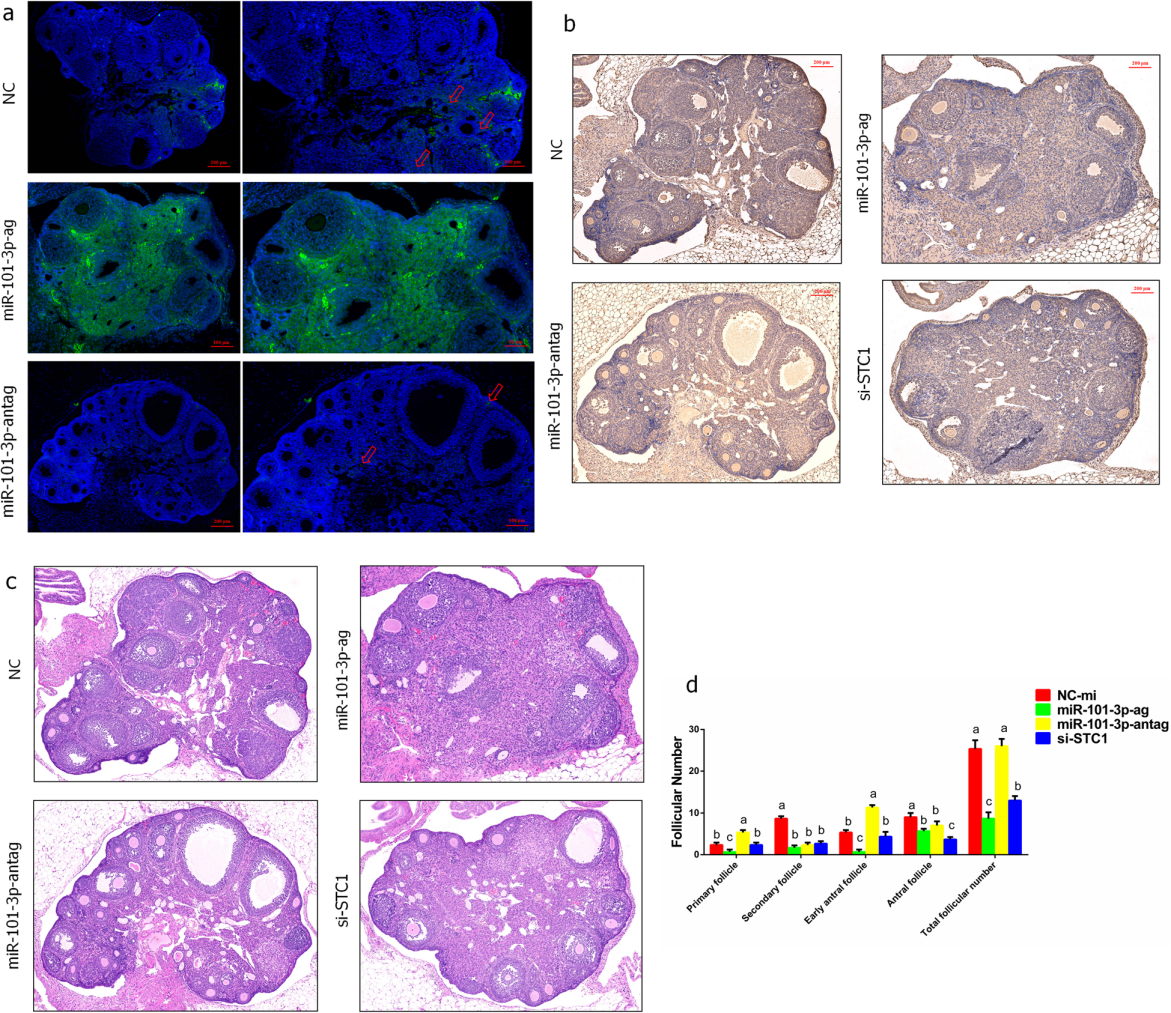
Effects of miR-101-3p and *STC1* on ovarian development in mice. Twelve mice are divided into four groups and treated with NC (100 μL saline), miR-101-3p agonists (miR-101-3p-ag; 10 nmol per time, with 100 μL saline), miR-101-3p antagonist (miR-101-3p-antag; 20 nmol per time, with 100 μL saline) or siRNA-STC1 (si-STC1; 20 nmol per time, with 100 μL saline). **a** FISH is performed to identify the distribution and expression levels of miR-101-3p in mouse ovaries. The nucleus stained by DAPI is blue under ultraviolet excitation, and the positive expression is green fluorescence of the corresponding fluorescein-labelled FAM. The red arrows represent a small amount of fluorescence of miR-101-3p. **b** Immunohistochemistry exhibits the expressions of *STC1* in dissected mouse ovaries. SABC-positive cells are dyed in brown, while cell nuclei are dyed with DAPI in blue. The area of positive cells (Area), mean optical density (Mean Density), and integrated optical density (IOD) are effective indicators for semi-quantitative analysis of immunohistochemistry results. **c** The mouse ovarian morphology is observed using HE staining. The sections are stained with hematoxylin in blue purple, and stained with eosin in red. **d** After HE staining, the follicles are counted at each stage. Follicle counting principles are as follows: primordial follicles: oocytes surrounded by a layer of flat granulosa cells or mixed cells of flat and cubic granulosa cells (total cell number < 7); primary follicles: oocytes surrounded by more than 7 cubic granulosa cells; secondary follicles: oocytes surrounded by more than 2 layers granular cells; early antral follicles: oocytes surrounded by 2–4 layers granulosa cells and contained a follicular antrum (diameter < 20 μm); antral follicles: follicles with an obvious follicular antrum. Red lines represent scale bars. Values are expressed as mean ± SD of *n* = 3. Superscripts (a, b, c) show significant difference

Then we confirmed whether miR-101-3p functions on mammalian ovarian physiological activities *in vivo*. The distribution and expression levels of miR-101-3p in mouse ovaries were identified through FISH. In NC group, miR-101-3p expressed in granulosa cells of primary, secondary follicles, theca cells, stroma cells, and corpus luteum partially (Fig. 10a). MiR-101-3p expressed in most regions of the ovary in miR-101-3p-ag group, incorporating theca cells, granulosa cells of primordial, primary, secondary, mature and growing follicles, corpus luteum and stroma cells (Fig. 10a). MiR-101-3p expression in the ovary was marginal, and only a small amount of fluorescence was observed in the local stroma cells in miR-101-3p-antag group (Fig. 10a). Immunohistochemistry exhibited that *STC1* expressed in theca cells and granulosa cells of follicles widely. MiR-101-3p overexpression and *STC1* depletion suppressed *STC1* expression in the ovaries, whereas miR-101-3p-antag treatment promoted *STC1* expression (Fig. 10b, Table 2). We then observed the morphology and counted follicles numbers at each stage after treated mice ovaries were stained with HE. Small and stunted ovarian fragments were observed in miR-101-3p-ag and si-STC1 groups, while miR-101-3p inhibition exhibited an opposite result (Fig. 10c). The results showed decreased numbers of primary, secondary, early antral, antral and total follicles in miR-101-3p-ag group, decreased numbers of secondary, antral and total follicles in si-STC1 groups (Fig. 10c, d). Increased numbers of primary and early antral follicles and decreased numbers of secondary and antral follicles were exhibited in miR-101-3p-antag group compared with NC (Fig. 10c, d).

**Table 2:** Identification of *STC1* expression in mouse ovary by immunohistochemical

Index		*STC1*			
		NC	miR-101-3p-ag	miR-101-3p-antag	si-STC1
Area	Mean	410.69	224.44	290.36	177.33
	SUM	350,725	244,639	304,008	252,695
Mean deneity	Mean	0.12	0.12	0.17	0.10
	SUM	105.10	130.78	175.31	143.69
IOD	Mean	58.06	30.56	53.71	20.47
	SUM	49,585.84	33,311.77	56,240.16	29,175
IOD/Area	SUM	0.1413	0.1361*	0.1849**	0.1154*

Area: the area of positive cells, Mean density: mean optical density, IOD: integrated optical density. * *P* < 0.05, ** *P* < 0.01, *n* = 3 independent experiments (mean ± SD)

## Discussion

Ovarian activity is the key to a successful reproduction [1]. The complex changes in tissue components and functions require a high degree of spatiotemporal synergy in proliferation, apoptosis and differentiation of diverse cells in follicle, corpus luteum and ovarian stroma [2]. Studies indicate that miRNAs take crucial effects on ovarian activities by regulating genes related to ovarian development [7–15]. Our previous work shows that miR-101-3p expresses differentially in dairy goat ovaries compared single with multiple litter sizes, indicating it may influence ovarian development (not published). Hence, more investigations are needed to recognize the specific functions of miR-101-3p on goat ovaries.

Firstly, our present work chose goat granulosa cells to study the potential molecular functions of miR-101-3p, since granulosa cells of ovarian follicles participate in oocyte nourishing, secreting steroid hormones that regulate ovarian function [2, 3]. Herein, a complete transcriptome dataset detailing the differentially expressed unigenes after miR-101-3p overexpression in granulosa cells was accomplished using RNA-Seq approach. A total of 142 DEGs was identified compared miR-101-3p mimics with NC groups, with 78 down-regulated genes including *C4BPA, STC1, EDN2, KCP* and *PDZK1* and 64 up-regulated genes including *FSHB, BMPER, CNNM1, NFIB* and *NR4A3*. *C4BPA* suppresses complement activation by binding to C4B and regulates lipid metabolism, inflammation and coagulation pathways [33]. *STC1* is recently reported to participate in a variety of reproductive-related processes, including ovarian growth and development [22–26]. *EDN2* is proposed to accelerate ovulation as a granulosa cell-derived contractile signal [34]. *KCP* interacts with BMPs and the BMP type I receptor, thus facilitates BMP signaling in a paracrine manner [35]. *PDZK1* interacts with vital membrane-associated proteins and transporters and is identified as an estrogen-regulated gene [36]. *FSHB*, as one of the follicle stimulating hormone (FSH) subunits, regulates crucial reproductive functions such as steroid production and ovarian development via FSH signaling [37]. *BMPER* is a BMP-binding endothelial cell precursor-derived regulator and involved in various cell biology by BMP signaling [38]. *CNNM1* works in self-renewal of stem cells of spermatogonia and cell cycle regulation [39]. *NFIB* belongs to transcription factors, implicated in cell differentiation, growth and other processes [40]. *NR4A3* regulates the transcription of overlapping target genes implicated in a series of cellular processes and works as a nuclear receptor [41]. This study also expanded the amount of genetic information available and provided a profile of physiological processes of these DEGs. Neuroactive ligand-receptor interaction, natural killer cell mediated cytotoxicity, cytokine-cytokine receptor interaction and complement and coagulation cascades were among the significantly enriched pathways. Our results furnish a first step toward a modified understanding of the functions of miR-101-3p on goat granulosa cells.

Next, we specialized in the specific effects of miR-101-3p on goat granulosa cells *in vitro*. From detected DEGs, we selected *STC1* for further research for the following reasons: (1) there is a miR-101-3p binding site of STC1-3′-UTR, (2) *STC1* is involved in the most significant down-regulated DEGs and (3) *STC1* is considered as a vital ovarian regulator. *STC1* shows a gestational and nursing state function, identifies cholesterol or lipid storage droplets which were subcellular luteal cell targets in ovaries [23, 24]. *STC1* also inhibits FSH-, LH- and hCG-stimulated progesterone synthesis in rat granulosa cells and bovine luteal cells [25, 26]. In swine ovarian follicles, *STC1* acts as a physiological regulator of follicular function and modulates redox status in granulosa cells [42]. Our work showed that miR-101-3p targeted the 3′-UTR of *STC1* and further blocked its mRNA and protein levels, revealing that miR-101-3p specifically targets *STC1*. Different steroid hormones affect follicular development via granulosa cell growth and follicular fluid formation, including cell proliferation, apoptosis and angiogenesis within the follicle. E2 and P4 are well-known steroid hormones and regulate the expression of related genes involved in ovulation and luteal formation [31]. Thus this study detected the effects of miR-101-3p and *STC1* on E2 and P4 and showed that miR-101-3p promoted and *STC1* suppressed E2 and P4 secretions. We also found that miR-101-3p increased *CYP11A1* and *3β-HSD* mRNA levels and STAR, CYP19A1 and 3β-HSD protein levels but decreased CYP11A1 protein levels. *STC1* promoted the protein expressions of STAR, CYP11A1, CYP19A1 and 3β-HSD. Thus we speculate that miR-101-3p and *STC1* affect E2 and P4 secretions via *STAR, CYP11A1, CYP19A1* and *3β-HSD* steroid hormone synthesis-associated genes. Co-expression of miR-101-3p with *STC1* exhibited an adiaphorous effect on E2 and P4 secretions and CYP11A1, CYP19A1, 3β-HSD and STAR expressions. Therefore, the regulation of miR-101–3p on steroid hormone synthesis in granulosa cells partly relies on *STC1*.

The proliferation and apoptosis of granulosa cells are closely related to ovarian development [2, 3]. Accordingly, we explored the mechanism of miR-101-3p and *STC1* in cellular survival capabilities of goat granulosa cells. The study revealed that miR-1013p inhibited, while *STC1* promoted granulosa cell proliferation. Co-expression of miR-101-3p with *STC1* indicated a neutral effect. Our data also showed that miR-101-3p inhibited CDK4, CCND1, CCNE1 and PCNA expressions while *STC1* inhibition blocked CDK4 and CCND1 protein expressions. *STC1* overexpression accelerated CKD4, CCND1 and PCNA but restrained CCNE1 protein levels, and mitigated the miR-101-3p’s role on CCND1, CCNE1 and PCNA. These findings indicate that miR-101-3p inhibits granulosa cell proliferation by regulating *CDK4, CCND1, CCNE1* and *PCNA* proliferation-related genes via *STC1*. FCM assay demonstrated that miR-101-3p promoted and *STC1* inhibited the apoptotic rates of granulosa cells. MiR-101-3p induced pro-apoptotic Bax and p53 expressions and reduced anti-apoptotic Bcl-2 expression. *STC1* decreased the expressions of Bax, p53 and pro-apoptotic Caspase3 and restrained that of Bcl-2. Moreover, *STC1* partly alleviated the effects of miR-101-3p on granulosa cell apoptotic rates and Bcl-2, Bax, p53 expressions. Thus, we speculate that miR-101-3p promotes granulosa cell apoptosis by regulating *Bcl-2, Bax, p53* and *caspase3* via *STC1* depletion.

PI3K-AKT-mTOR signalling pathway has an imperative role on protein synthesis and cellular processes including proliferation and apoptosis through co-regulated proteins [32]. *PI3K* is a key upstream motivator of *AKT*. *AKT* can give rise to *mTOR* phosphorylation, which also mediates metabolism processes to maintain cell growth and proliferation [43]. The tumour-suppressor *PTEN* is a vital passive mediator of cell-survival signalling pathways initiated by *PI3K* [44]. It’s also reported the impacts of miR-101-3p on PI3K-AKT pathway in various kind of cells. In Saos-2 cells, miR-101 transfection inhibits the mRNA and protein expressions of mTOR, which consequently improves cell apoptosis and suppresses cell proliferation [16]. MiR-101 overexpression disrupts the PI3K-AKT pathway and promotes Bcl2-regulated apoptosis induced by *RLIP76* in prostate cancer cells [17]. MiR-101 represses tumour growth and migration by down-regulating *ROCK1* and inactivating PI3K-AKT and JAK-STAT pathways in osteosarcoma cells [18]. According to these reports, we explored whether miR-101-3p and *STC1* could affect PI3K-AKT-mTOR pathway in goat granulosa cells. Our results showed that miR-101-3p inhibited PI3K, AKT and mTOR but enhanced PTEN protein levels. MiR-101-3p also triggered the activation of AKT and mTOR. We observed increasing PI3K, AKT, mTOR and p-mTOR expressions and decreasing PTEN expression after *STC1* was induced. The effects of miR-101-3p on motivation and expression of these key proteins were partially alleviated via *STC1*. These findings support that miR-101-3p can regulate granulosa cell proliferation and apoptosis through *STC1* by inhibiting the PI3K-AKT-mTOR pathway.

The above experiments show that miR-101-3p can regulate biological processes of goat granulosa cells cultured *in vitro* through the target gene *STC1*. However, whether miR-101-3p affects ovarian physiological activities consistently *in vivo* requires further research. Therefore, mouse ovaries were used to study miR-101-3p functions. Mice were randomly divided into four groups and were injected with intraperitoneal drugs. FISH results showed that miR-101-3p expressed or marginal expressed in most regions of the ovaries in miR-101-3p-ag or -antag groups. Hence, miR-101-3p agonists and antagonists were efficient and available for further research. We demonstrated that miR-101-3p overexpression and *STC1* depletion inhibited, whereas miR-101-3p inhibition promoted *STC1* expression in mouse ovaries, indicating that miR-101-3p inhibits *STC1* in mouse ovaries. Moreover, miR-101-3p exhibited unusual ovarian development functions, as reflected by small and stunted ovarian fragments and decreased numbers of various follicles. We also observed consistent results after *STC1* was inhibited. Thus we speculate that miR-101-3p disrupts ovarian development in mouse ovaries and may partly performs its effects by *STC1*. The *in vivo* experiment results showed that the developmental status of mouse follicles coincided with miR-101-3p promoting apoptosis and inhibiting proliferation of granulosa cells *in vitro*, indicating that miR-101-3p may regulate ovarian development in dairy goats. Although, big species differences are present between dairy goat and mouse, miRBase database (http://www.mirbase.org/) shows that the seed sequences of chi-miR-101-3p and mmu-miR-101-3p are similar (Fig. S3). The seed sequences (2–8 nts) which located at 5′ end of miRNAs, can pair with the 3′-UTR of their target genes and then trigger mRNA or/and protein degradation. The miRNA binding sites are conserved in multiple species [4, 5]. Hence, the highly conserved structure and function of miRNAs demonstrate the feasibility of our study using acknowledged model animals *in vivo*.

## Conclusion

In conclusion, 78 down-regulated and 64 up-regulated DEGs were identified after miR-101-3p overexpression in goat granulosa cells using RNA-Seq. GO terms and KEGG pathway analysis demonstrated that DEGs could participate in the regulation of ovarian growth and development. *In vitro*, miR-101-3p targeted *STC1,* one of down-regulated DEGs directly and inhibited its expressions in goat granulosa cells. MiR-101-3p induced E2 and P4 secretions by *STAR, CYP19A1, CYP11A1* and *3β-HSD* steroid hormone synthesis-associated genes partially via *STC1* depletion. MiR-101-3p also inhibited the proliferation and promoted the apoptosis of granulosa cells by regulating PI3K-AKT pathway key genes *PI3K, PTEN, AKT* and *mTOR* via *STC1* depletion. *In vivo*, miR-101-3p inhibited *STC1* expression and ovarian development, while *STC1* promoted ovarian development in mouse ovaries. Our results provided a theoretical basis and experimental evidence for miR-101-3p functions on goat ovarian development.

## Supplementary information

**Additional file 1: Figure S1.** The transfection efficiency of miR-101-3p and *STC1.*

**Additional file 2: Figure S2.** High-throughput sequencing of RNA

**Additional file 3: Figure S3.** The seed sequences of miR-101-3p in goat and mouse.

**Additional file 4: Table S1.** The sequences of miR-101-3p mimics, 101-3p inhibitors, NC, inhibitor NC and si-STC1.

**Additional file 5: Table S2.** The validated primers used for RT-PCR.

**Additional file 6: Table S3.** Distribution of effective sequences in reference genomes.

**Additional file 7: Table S4.** The up- and down-regulated genes in the miR-101-3p mimics VS negetive control (NC) groups.

**Additional file 8: Table S5.** GO enrich of differentially expressed genes.

**Additional file 9: Table S6.** Pathway annotations of differentially expressed genes.

## Data Availability

The datasets used and/or analyzed during the current study are available from the corresponding author on request.

## Abbreviations

DEGs: Differentially expressed unigenes
E2: Estrogen
P4: Progesterone
FISH: Fluorescence *in situ* hybridisation
HE: Hematoxylin-eosin
nts: Nucleotides
UTR: Untranslated region
*STC*: Stanniocalcin
RNA-Seq: High-throughput sequencing of RNA
FBS: Foetal bovine serum
RPKM: Reads per kilobase transcriptome per million mapped reads
GO: Gene Ontology
RT-qPCR: Quantitative real-time PCR
ELISA: Enzyme-linked immunosorbent assay
FCM: Flow cytometry assay
PI: Propidium iodide
CAMs: Cell adhesion molecules
CDS: Coding sequence
FSH: Follicle stimulating hormone
