# Supplementary figures and images for: Effects of miR-101-3p on goat granulosa cells in vitro and ovarian development in vivo via STC1
Source: J Anim Sci Biotechnol. 2020 Oct 14;11:102. doi: 10.1186/s40104-020-00506-6 (PMC7557009; doi:10.1186/s40104-020-00506-6)

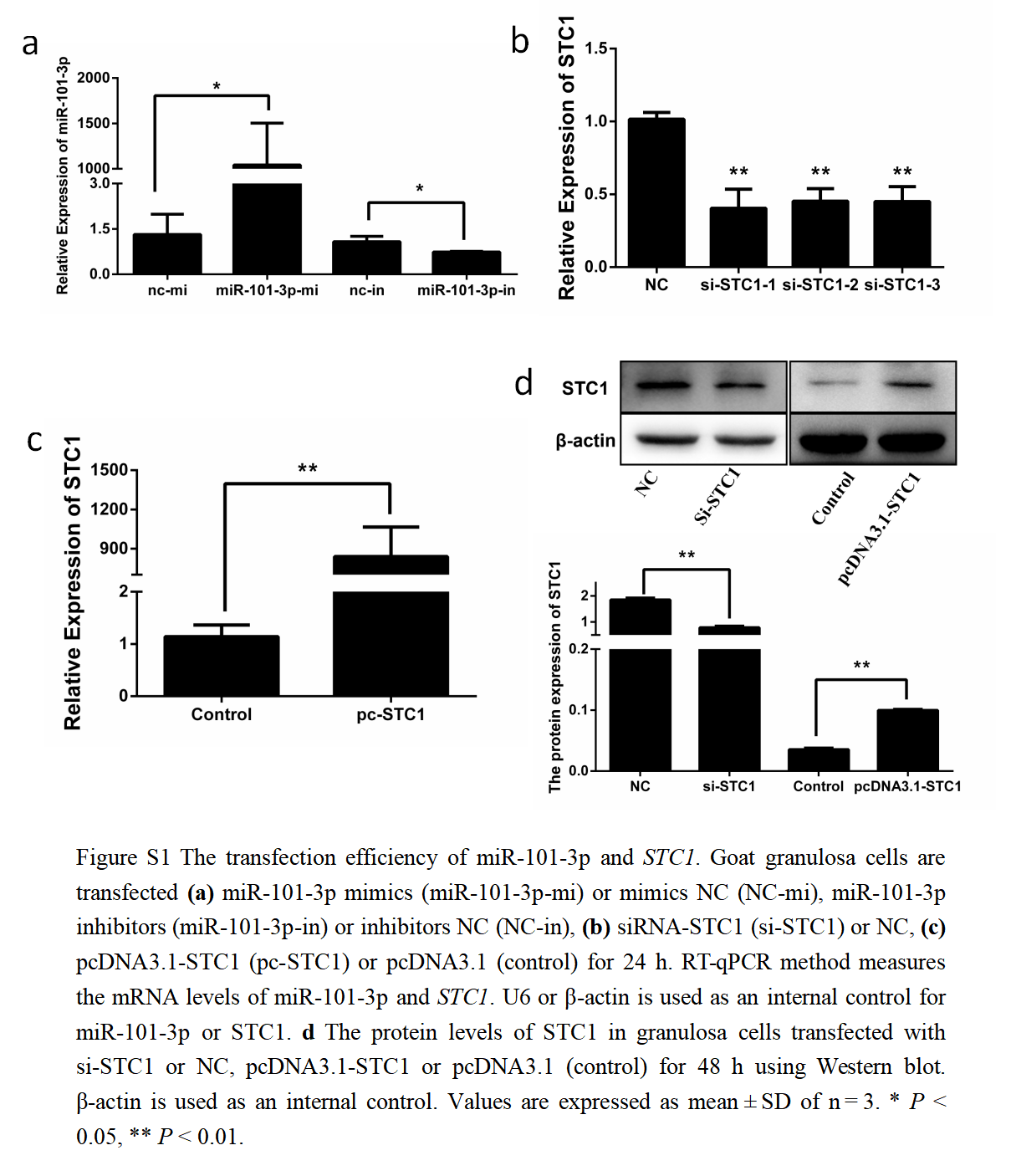

Supplement: Supplementary file 1 — Additional file 1: Figure S1. The transfection efficiency of miR-101-3p and STC1. [file 40104_2020_506_MOESM1_ESM.tif]

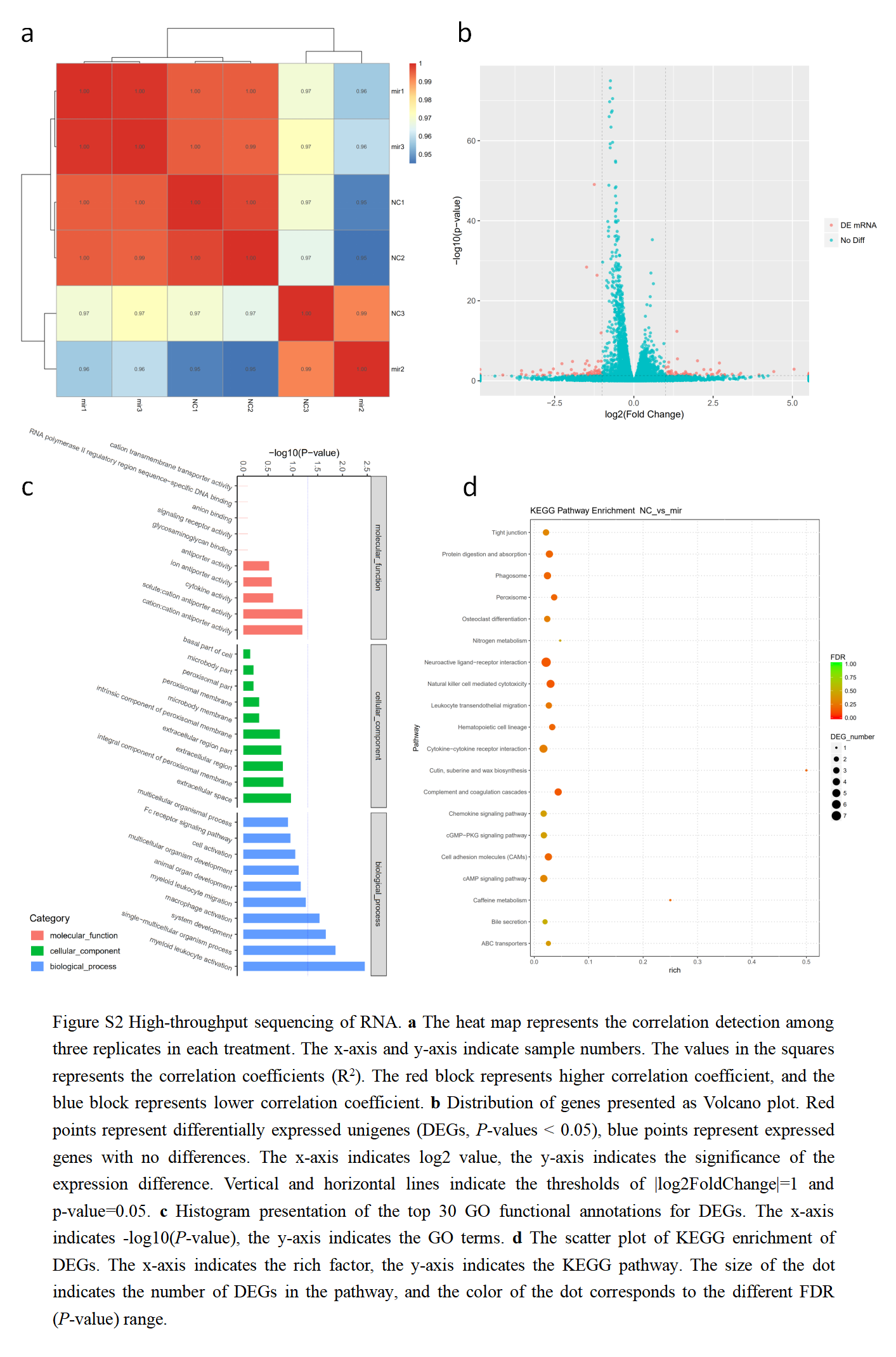

Supplement: Supplementary file 2 — Additional file 2: Figure S2. High-throughput sequencing of RNA [file 40104_2020_506_MOESM2_ESM.tif]

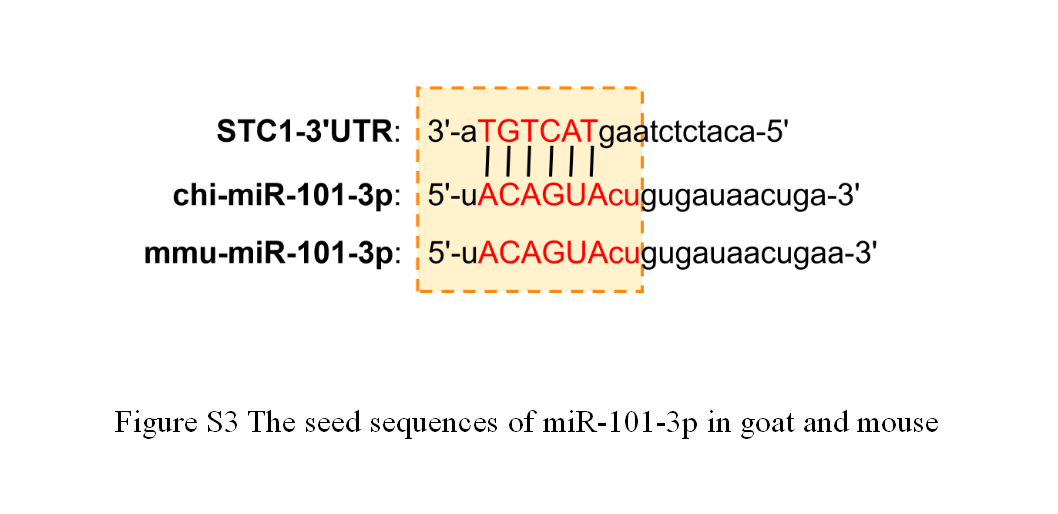

Supplement: Supplementary file 3 — Additional file 3: Figure S3. The seed sequences of miR-101-3p in goat and mouse. [file 40104_2020_506_MOESM3_ESM.tif]
