# Supplementary material for: Effects of miR-101-3p on goat granulosa cells in vitro and ovarian development in vivo via STC1
Source: J Anim Sci Biotechnol. 2020 Oct 14;11:102. doi: 10.1186/s40104-020-00506-6 (PMC7557009; doi:10.1186/s40104-020-00506-6)
Supplement: Supplementary file 4 — Additional file 4: Table S1. The sequences of miR-101-3p mimics, 101-3p inhibitors, NC, inhibitor NC and si-STC1. [file 40104_2020_506_MOESM4_ESM.doc]

Table S1 The sequences of miR-101-3p mimics, miR-101-3pinhibitors, NC, inhibitor NC and si-STC1

| Name | Sequence (5’-3’) |
| --- | --- |
| NC | CAGUACUUUUGUGUAGUACAA |
| miR-101-3p mimics | UACAGUACUGUGAUAACUGA |
| inhibitor NC | UUCUCCGAACGUGUCACGUTT |
| miR-101-3p inhibitors | UCAGUUAUCACAGUACUGUA |
| si-STC1 | GCAUUUGUCAAAGAGAGCUTT |
