# Supplementary material for: Effects of miR-101-3p on goat granulosa cells in vitro and ovarian development in vivo via STC1
Source: J Anim Sci Biotechnol. 2020 Oct 14;11:102. doi: 10.1186/s40104-020-00506-6 (PMC7557009; doi:10.1186/s40104-020-00506-6)
Supplement: Supplementary file 5 — Additional file 5: Table S2. The validated primers used for RT-PCR. [file 40104_2020_506_MOESM5_ESM.doc]

Table S2 The validated primers used for RT-PCR

| Gene name | Primer | Primer sequences (5'-3') | Product size (bp) |
| --- | --- | --- | --- |
| 3β-HSD | Forward | TCTGCCTGTTGGTGGAGGAGAAG | 200 |
|  | Reverse | GATGACAGAAGCGGTGTGGATGAC |  |
| ADAMTS15 | Forward | TGACGCCAGATGCTCAGTTC | 213 |
|  | Reverse | TGACGCCAGATGCTCAGTTC |  |
| BAX | Forward | CATCAACTGCCTTGGACTTT | 130 |
|  | Reverse | GACCACTCCTCCCTACCCT |  |
| BCL2 | Forward | AGAGCGTCAACCGGGAGATG | 167 |
|  | Reverse | CAGCCAGGAGAAATCAAACAGG |  |
| BMPER | Forward | ACCTGCTGCGTCTTGCTG | 111 |
|  | Reverse | CTGGAGGACTTCACCTTCATTT |  |
| C1QC | Forward | GGATGGGTATGACGGACTGC | 220 |
|  | Reverse | CGACTGGTGCTTCTGCTTGT |  |
| C4BPA | Forward | GGCGTCCTAGAACACCAGAATGC | 184 |
|  | Reverse | GGTGACCAGCCTGAAGAAGTGC |  |
| Caspase 3 | Forward | ATACCAGTTGAGGCAGAC | 161 |
|  | Reverse | TTAACCCGAGTAAGAATGT |  |
| CCDC33 | Forward | ACCGTGAGGCACCAGGAGAA | 159 |
|  | Reverse | GCCCGTAGGACCCAGAGGAA |  |
| CCND1 | Forward | TTCCTCTCCTATCACCGCCTGAC | 173 |
|  | Reverse | TCCTCCTCTTCCTCCTCCTCCTC |  |
| CCNE1 | Forward | AAGTGCTCCTGCCTCAGTATCCTC | 125 |
|  | Reverse | ATACAAGGCGGAAGCAGCAAGTAC |  |
| CDK4 | Forward | GCTGCTGCTGGAGATGCTGAC | 100 |
|  | Reverse | CTCTGCGTCACCTTCTGCCTTG |  |
| CLEC9A | Forward | ATGTTTTCCCAGGGGTGTGAAAAGG | 147 |
|  | Reverse | CCTTTTCACACCCCTGGGAAAACAT |  |
| CNNM1 | Forward | GTGGAGGTGGAGGTTGGTAA | 112 |
|  | Reverse | CCTTCCGCACATCGTTATCT |  |
| CYP19A1 | Forward | AGCATCTGGACAGGTTGGAGGAG | 181 |
|  | Reverse | TTGAGAAGGAGAGCTTGCCATGC |  |
| CYP11A1 | Forward | GCTGCGGAAGGAGGTTCTGAATG | 194 |
|  | Reverse | GCACCAGTGTCTTGGCAGGAATC |  |
| FSHB | Forward | GTTATGTCCCTCCTTGTGC | 127 |
|  | Reverse | GCTTGGCTATCTTGGTGTC |  |
| GUCY2C | Forward | CCTGCTGCTTCTCATTGCTCTCC | 172 |
|  | Reverse | CTGGATTGTGTCTCGCCTCTTGTC |  |
| IRF8 | Forward | AGGCAGGGCGTGCTGGTCAA | 136 |
|  | Reverse | GAAAGAACTGGCTGGTGTCG |  |
| KRT7 | Forward | CTTCGGCAGCAGCAGCAGTC | 122 |
|  | Reverse | GCAGGCTCTGATTGATGGTGACC |  |
| LOC102178901 | Forward | GCTTTCGCAATGTCTGGTCG | 106 |
|  | Reverse | GCTTGGTAATGCCCTGGATG |  |
| LOC102185049 | Forward | GTGGCAAGCAAGGCGGTAAGG | 179 |
|  | Reverse | AATCTCGGCGGTCAGATACTCCAG |  |
| LOC102189835 | Forward | AGCTGCCACGGTTGGATTATTGG | 90 |
|  | Reverse | ACTCTTCAGAGCAACACCAGCAAC |  |
| MCOLN3 | Forward | CAATCTCCTCATTCTCACCCTC | 110 |
|  | Reverse | CCAGCACGATCCATCCAC |  |
| NDUFA4L2 | Forward | GCTTCTACCGGCAGATCAAGAGAC | 103 |
|  | Reverse | AAGGCAAGTCGCAGCAAGTAGAG |  |
| P53 | Forward | CCCCTTCCCTCAACAAGC | 144 |
|  | Reverse | GCCTCACAACCTCCGTCA |  |
| PCNA | Forward | GTAGCCGTGTCATTGCGACTCC | 145 |
|  | Reverse | GCTCTGTAGGTTCACGCCACTTG |  |
| RETN | Forward | CCTTCCTCTTCATCCCAGTC | 137 |
|  | Reverse | GTCCAGGCCAATGTTCCTTA |  |
| S100A9 | Forward | CGGCTAAGGGACCCGGAAAC | 180 |
|  | Reverse | GGCCACCAGCATAATGAACT |  |
| SLC11A1 | Forward | TCGCCCTGTCTGTCTCCTTC | 184 |
|  | Reverse | ATCACGCCTCCTTGGTAAAT |  |
| STAR | Forward | GCGACCAAGAGCTTGCCTATATCC | 106 |
|  | Reverse | TTGGCCTGCCGACTCTCCTTC |  |
| STC1 | Forward | AGCCTACTGGACTGTGACGAAGAC | 159 |
|  | Reverse | CTCATTGGCACGCCTCCTGTTG |  |
| miR-101-3p | RT primer | GTCGTATCCAGTGCAGGGTCCGAGGTATTCGCACTGGATACGACTCAGTT |  |
|  | Forward | TTG CGG CGG TAC AGT ACT GTG AT |  |
|  | Reverse | ATCCAGTGCAGGGTCCGAGG |  |
| β-actin | Forward | GGGTCATCTTCTCACGGTTG |  |
|  | Reverse | GATCTGGCACCACACCTTCT |  |
| U6 | Forward | GTG CTC GCT TCG GCA GCA CAT |  |
|  | Reverse | ATCCAGTGCAGGGTCCGAGG |  |
