# Supplementary material for: Effects of miR-101-3p on goat granulosa cells in vitro and ovarian development in vivo via STC1
Source: J Anim Sci Biotechnol. 2020 Oct 14;11:102. doi: 10.1186/s40104-020-00506-6 (PMC7557009; doi:10.1186/s40104-020-00506-6)
Supplement: Supplementary file 6 — Additional file 6: Table S3. Distribution of effective sequences in reference genomes. [file 40104_2020_506_MOESM6_ESM.doc]

Table S3 Distribution of effective sequences in reference genomes

| Sample | Useful reads | Mapped to Gene Count（%） | Mapped to Inter Gene Count（%） | Mapped to Exon Count（%） |
| --- | --- | --- | --- | --- |
| NC1 | 80375164 | 71567439（89.04%） | 8807725（10.96%） | 62907762（87.90%） |
| NC2 | 69725503 | 61722082（88.52%） | 8003421（11.48%） | 54223270（87.85%） |
| NC3 | 73527376 | 60893557（82.82%） | 12633819（17.18%） | 51621385（84.77%） |
| miR-101-3p-1 | 75181716 | 66373318（88.28%） | 8808398（11.72%） | 57407773（86.49%） |
| miR-101-3p-2 | 85678635 | 67900657（79.25%） | 17777978（20.75%） | 55726693（82.07%） |
| miR-101-3p-3 | 67769468 | 59212742（87.37%） | 8556726（12.63%） | 50555674（85.38%） |
